# From neural loss to regeneration: modulating cell death to enhance pluripotent stem cell graft survival and integration

**DOI:** 10.1038/s41419-026-09066-5

**Published:** 2026-07-17

**Authors:** Ouye Chen, Kelvin K. Hui, Fabian Raudzus, Jeffrey T. Henderson

**Affiliations:** 1 Graduate Department of Pharmaceutical Sciences, Faculty of Pharmacy, University of Toronto, Toronto, ON Canada; 2 Center for iPS Cell Research and Application (CiRA), Kyoto University, Kyoto, Japan; 3 Neuronal Signaling and Regeneration Unit, Graduate School of Medicine, Kyoto University, Kyoto, Japan; 4 Center for Medical Education and Internationalization (CMEI), Graduate School of Medicine, Kyoto University, Kyoto, Japan

**Keywords:** Regeneration and repair in the nervous system, Translational research, Cell death in the nervous system, Regeneration, Cell death

## Abstract

Cerebrovascular and neurodegenerative diseases rank among the leading causes of death worldwide and represent an increasing socioeconomic burden, particularly in aging populations. Pluripotent stem cell (PSC)-based therapies have emerged as promising strategies for replacing lost neurons and restoring neural circuits in disorders of the central nervous system (CNS). However, major barriers remain, including poor survival, limited integration, and variable functional maturation of transplanted cells. These challenges currently limit the reproducibility and clinical translation of PSC-based therapies. Here, we examine these barriers with a particular focus on ischemic stroke and Parkinson’s disease, two conditions that represent complementary models of acute and chronic neuronal loss and are among the most advanced indications for PSC-based transplantation. We discuss how programmed cell death signaling contributes not only to neuronal loss in these disorders but also to the limited survival of transplanted PSC-derived grafts. Building on this mechanistic framework, we highlight strategies that may improve graft survival and functional integration. Specifically, we propose an integrated approach that combines modulation of programmed cell death pathways, targeted activation of RAS signaling, optimization of mitochondrial health, and use of biocompatible scaffolds to support neuronal maturation and network integration. Together, these strategies provide a conceptual framework for improving the reliability and therapeutic efficacy of PSC-based cell transplantation therapies and accelerating their translation toward clinical application.

**Figure:**
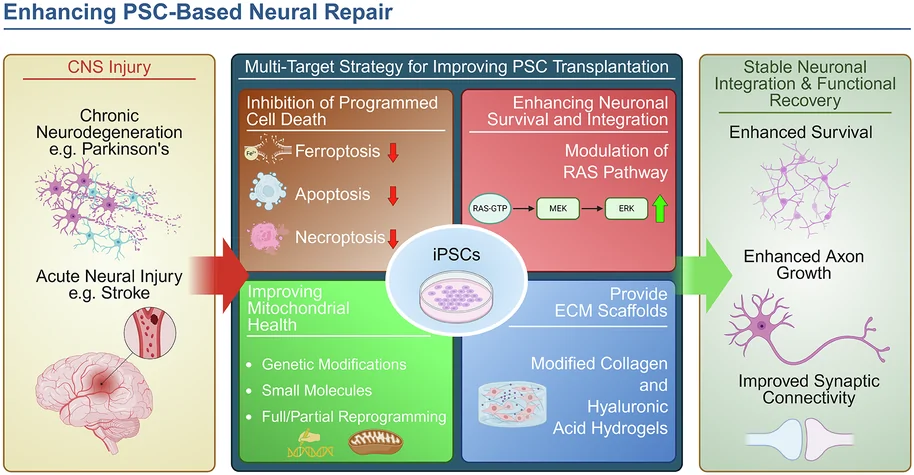
Overview of strategies to enhance PSC-derived neural graft survival and integration through modulation of cell death pathways, RAS signaling, mitochondrial function, and extracellular scaffolds. Created in BioRender. Raudzus, F. (2026) https://BioRender.com/blr0ycj.

Overview of strategies to enhance PSC-derived neural graft survival and integration through modulation of cell death pathways, RAS signaling, mitochondrial function, and extracellular scaffolds. Created in BioRender. Raudzus, F. (2026) https://BioRender.com/blr0ycj.

## Facts

- Neuronal damage resulting from acute neural injuries and chronic neurodegenerative diseases represents a major and increasing socioeconomic burden, particularly in aging populations.
- Stem cell–based approaches for CNS repair have demonstrated encouraging safety profiles and potential efficacy in preclinical and early clinical studies; however, variability in graft survival, integration, and dosing remains a significant challenge. Improving the neuronal survival and integration of implants is required for more effective stem cell–based therapies.
- Animal developmental studies and injury/disease models have shown that the involvement of multiple programmed cell death (PCD) pathways contributes to neuronal loss, with substantial convergence on mitochondrial signaling mechanisms.
- Inhibition of PCD and modulation of H-RAS signaling have been reported to promote neuronal survival and connectivity in animal studies, such that a combinatorial approach promises to advance current stem cell–based therapies for reproducible, widespread applications.

## Introduction

Neurological disorders affecting the central nervous system (CNS) have been identified as the leading source of total disability in adjusted life-years and rank third behind cardiovascular diseases and cancer [1], and are specifically accelerating in countries with aging populations. Acute neural injuries, such as stroke and spinal cord injury, together with neurodegenerative disorders, including Alzheimer’s and Parkinson’s diseases (AD and PD, respectively), represent a growing strain on healthcare systems around the globe. In the past 30 years, the absolute incidence of stroke has increased by 70%, with more than 12.2 million cases in 2019 [2]. Stroke is broadly divided into two major types: ischemic, resulting from the obstruction of cerebral blood flow, or hemorrhagic, in which the leakage or rupture of cerebral vessels induces downstream cellular injury. Currently, more than 85% of clinical strokes are of the ischemic subtype [3], though hemorrhagic strokes exhibit a 4-fold higher rate of lethality. Among neurodegenerative disorders, AD and PD exhibit the highest prevalence and mortality, followed by Huntington’s disease, amyotrophic lateral sclerosis, and frontotemporal dementia, all of which are rising within the aging population [4].

While efforts continue worldwide to gain insight into the molecular mechanisms governing AD and PD pathogenesis, without better biomarkers and predictive tools, most clinical cases are diagnosed only when severe neural damage has already been inflicted. In contrast to other organ systems, such as the liver and even the peripheral nervous system (PNS), the CNS exhibits a severely limited intrinsic capacity for regeneration, thereby requiring alternative therapeutic approaches, including neural augmentation in the form of stem cell replacement.

Understanding of the primary neural circuitry affected in these disorders suggests that targeted neural augmentation could, in principle, provide significant clinical improvement, as demonstrated by experimental studies in animal models [5, 6]. Pluripotent stem cell (PSC)-based replacement therapies have therefore been explored experimentally and have shown encouraging safety profiles and signs of clinical benefit in early trials [7, 8]. However, it has become increasingly clear that substantial improvements, particularly regarding cellular integration and survival, are required before these approaches can achieve reliable therapeutic outcomes. Indeed, both historical and recent transplantation studies have identified graft survival and functional integration as fundamental roadblocks to reproducibility between individuals and overall clinical success.

The localized nature of neural insults in PD, particularly during its early stages, and in ischemic stroke, together with the relatively well-defined neuronal populations affected, makes these disorders especially suitable targets for cell replacement strategies. Both are widely regarded as leading candidates for PSC-based therapies, as reflected by their comparatively advanced stage of clinical translation, due to: (i) the spatially defined nature of neuronal loss, (ii) the relative clarity of affected circuits, and (iii) the largely irreversible degeneration that renders endogenous repair insufficient.

In contrast to more diffuse neurodegenerative disorders such as Alzheimer’s disease, PD and stroke provide settings in which targeted neuronal replacement can integrate into existing circuitry and yield measurable functional recovery. Accordingly, we focus on PD and stroke as complementary examples of chronic neurodegeneration and acute neural injury that are currently undergoing intensive investigation for PSC-based cell therapies but share common challenges related to graft survival and integration. Understanding the molecular mechanisms underlying these barriers, particularly the role of programmed cell death pathways affecting both host tissue and transplanted cells, may therefore provide actionable strategies to improve transplantation outcomes.

In this review, we discuss how host and graft cell death limit PSC-based therapies and highlight mechanistically informed strategies to enhance graft survival and integration, with particular emphasis on PD and stroke, while also considering implications for other CNS injuries and neurodegenerative disorders.

## Repairing the damaged brain using PSC-based cell replacement therapy

Given the need to replace damaged or lost neurons and the limited capability of mature neurons to regenerate following injury or during disease, cell transplantation has been considered an alternative therapeutic approach for repairing the damaged brain and reestablishing neural connections. Grafting new neurons into the human brain, however, is understandably a complicated process. Historically, the transplantation of fetal ventral mesencephalic cells as a putative source of dopaminergic neurons for PD therapy started four decades ago. Despite years of effort and improvement, only limited success has been achieved owing to poor survival and insufficient axonal outgrowth of dopaminergic neurons [9–12]. With limited availability and ethical concerns being primary challenges for fetal brain tissue transplantations, PSC-based cell replacement therapies offer a more promising and viable treatment approach for neural injuries and neurodegenerative disorders.

Since the discovery of PSCs, capable of self-renewal and differentiation into various somatic cell types, there has been tremendous interest in leveraging them for cell therapies to replace damaged, diseased, or aging tissues. While attention has traditionally been focused on employing embryonic stem cells (ESCs) for medical applications, their use has been limited by ethical, social, and legal concerns. With the emergence of induced pluripotent stem cells (iPSCs) as a viable alternative to ESCs nearly two decades ago, PSC-based therapies are closer to reality than ever. Next, we will examine recent efforts to bring them to the bedside and the remaining challenges that must be resolved for their widespread use.

### ESCs as a cell source for PSC-based therapies

ESCs, isolated from the inner cell mass of human embryos, can, in theory, differentiate into any extra-placental cell types. Induction of neural stem/progenitor cells (NSCs or NPCs) and subsequent neuronal subtypes from ESCs has been widely used to study essential signaling events during early CNS development [13, 14]. With several key improvements in efficiency and survival during neural induction over the past decades [15–17], the possibility of using human ESC-derived NSCs and NPCs as a therapeutic approach has become more attractive. To this end, there has been growing evidence from animal studies demonstrating the feasibility of this approach. In 2002, the direct transplantation of mouse ESCs into 6-OHDA-induced PD model rats generated mesencephalic dopaminergic-like cells and reduced PD symptoms [18]. Later, either human or mouse ESC-derived NPCs showed better survival and engraftment in similar rodent models, suggesting that in vitro differentiation of ESCs to NPCs for PSC-based transplantation therapy not only reduces off-target differentiation but also enhances viability and integration of transplanted cells [15, 19]. Studies using non-human primate PD models to validate the clinical safety and benefits of transplanting ESC-derived dopaminergic neurons have similarly yielded positive results, further demonstrating the feasibility of this method [20, 21]. Similar approaches have been tested in various animal models of acute neural injuries, where monkey ESC-derived Islet1^+^ neurons were engrafted into cryogenically injured mice, with improvements in motor function observed in hemiplegic mice [22, 23].

Subsequently, several human ESC-based therapies have been tested in clinical trials for stroke and PD (Table 1). NCT04631406 was the first clinical trial testing the potential of ESC-derived NSCs to improve motor function after stroke. Eighteen patients were reported to have received the cell transplant, with most patients showing functional recovery by 12 months following transplantation and adverse events that resolved spontaneously, thus suggesting safety and efficacy [24]. Conversely, several clinical trials (NCT04802733, NCT05635409, and NCT05887466) testing the transplantation of dopaminergic progenitor cells for PD treatment have been reported [25]. Human ESCs were expanded and differentiated through a detailed procedure to obtain TH^+^ OTX2^+^ FOXA2^+^ dopaminergic progenitor cells for transplantation [25, 26]. Leveraging advances in dopaminergic precursor cell development, in vitro differentiation was carefully monitored by examining gene and protein expression. These preclinical studies revealed the benefits of transplanting partially differentiated ESCs to significantly improve motor functions in animal models while preventing teratoma formation and limiting engraftment generally to the targeted site.

**Table 1:** Current phase I/II clinical trials targeting acute neural injury (ischemic stroke) and chronic neurodegenerative disorders (Parkinson’s disease) using PSC-based approaches.

Clinical trial ID	Disease type	Cell type	Start date	Responsible parties	Countries	Planned participants
NCT03726814	Ischemic stroke	Peripheral blood-derived endothelial progenitors	12/2018	Allife Medical Science and Technology Co., Ltd.	China	12
NCT03725865	Ischemic stroke	Peripheral blood-derived neural stem cells	03/2019	Allife Medical Science and Technology Co., Ltd.	China	12
NCT04631406	Ischemic stroke	ESC-derived neural stem cell	01/2021	Stanford University	U.S.	30
NCT06299033	Ischemic stroke	iPSC-derived forebrain neural progenitor cells (allogeneic)	11/2023	Hopstem Biotechnology, Inc.	China	21
NCT02452723	Parkinson’s disease	Parthenogenetic stem cell-derived neural stem cells	07/2016	Cyto Therapeutics Pty Ltd.	Australia	12
NCT03119636	Parkinson’s disease	Parthenogenetic ESC-derived neural stem cells	05/2017	Chinese Academy of Sciences	China	50
UMIN000033564	Parkinson’s disease	iPSC-derived dopaminergic precursors (allogeneic)	08/2018	Kyoto University/Sumitomo Pharma Co.	Japan	7
NCT03815071	Parkinson’s disease	iPSC-derived neural stem cells (autologous)	02/2019	Allife Medical Science and Technology Co., Ltd.	China	10
NCT04802733	Parkinson’s disease	ESC-derived dopamine precursors	05/2021	BlueRock Therapeutics	U.S.	12
NCT05635409	Parkinson’s disease	ESC-derived dopamine precursors	11/2022	Lund University	Sweden/U.K.	8
NCT05897957	Parkinson’s disease	ESC-derived dopamine precursors	05/2023	BlueRock Therapeutics	U.S.	12
NCT05887466	Parkinson’s disease	ESC-derived dopamine precursors	05/2023	S.Biomedics Co., Ltd.	South Korea	12
NCT06145711	Parkinson’s disease	iPSC-derived dopaminergic precursors (autologous)	11/2023	Tongji University/XellSmart Biomedical Co., Ltd.	China	3
IRCT20160704028786N2	Parkinson’s disease	ESC-derived Dopaminergic Progenitors	12/2023	Iranian academic center for education culture and research	Iran	4
NCT06344026	Parkinson’s disease	iPSC-derived dopamine precursors (autologous)	01/2024	Aspen Neuroscience	U.S.	9
NCT06482268	Parkinson’s disease	iPSC-derived dopaminergic progenitors (allogeneic)	06/2024	University of California, San Diego	U.S.	7
NCT06422208	Parkinson’s disease	iPSC-derived dopaminergic neurons (autologous)	08/2024	Harvard University	U.S.	6
NCT06687837	Parkinson’s disease	iPSC-derived dopaminergic neurons (autologous)	12/2024	Massachusetts General Hospital	U.S.	8
NCT06167681/NCT06608355	Parkinson’s disease	iPSC-derived dopaminergic progenitors (allogeneic)	01/2024, 08/2024	iRegene Therapeutics Co., Ltd.	China	40, 6
NCT06753331	Parkinson’s disease	iPSC-derived dopaminergic progenitors (allogeneic)	12/2024	Sumitomo Pharma America, Inc.	U.S.	23
NCT06778265	Parkinson’s disease	iPSC-derived dopaminergic progenitors (allogeneic)	03/2025	Shanghai UniXell Biotechnology Co., Ltd	China	12
NCT06978920	Parkinson’s disease	iPSC-derived dopaminergic progenitors (allogeneic)	05/2025	Nuwacell Biotechnologies Co., Ltd.	China	48
NCT06821529	Parkinson’s disease	iPSC-derived dopaminergic progenitors (allogeneic)	05/2025	iCamuno Biotherapeutics Ltd.	China	12
NCT06482268	Parkinson’s disease	iPSC-derived dopaminergic progenitors (allogeneic)	06/2025	University of California, San Diego	U.S.	7
NCT07080775/NCT07166757	Parkinson’s disease	iPSC-derived neural progenitor cell (allogeneic)	07/2025 09/2025	XellSmart Bio-Pharmaceutical (Suzhou) Co., Ltd	China	12
NCT07102342/NCT07028632	Parkinson’s disease	iPSC-derived neural progenitor cell (allogeneic)	10/2025 06/2025	iRegene Therapeutics Co., Ltd.	U.S., China	5, 30
NCT07212088	Parkinson’s disease	iPSC-derived dopaminergic progenitors (allogeneic)	02/2026	iCamuno Biotherapeutics Ltd.	China	12

NCT04802733, accompanied by its 5-year follow-up study NCT05897957, offers one of the most promising results from PD clinical trials testing hESC-based therapies to date, with improvements in both low-dose (0.9 million cells) and high-dose cohorts (2.7 million cells) and no reports of serious adverse events [7]. Despite a relatively low number of transplanted cells (2.7 million) compared to similar trials, participants receiving the high dose, on average, experienced about 1.5 h of well-controlled symptoms per day more than the low-dose cohort, providing additional insights into the survival and integration efficiency of this approach.

### iPSCs as a more applicable ESC alternative

iPSCs, similar to ESCs in their self-renewal and differentiation abilities, can be reprogrammed from somatic cells by introducing *POU5F1* (encoding OCT3/4), *SOX2*, *c-MYC*, and *KLF4* (also known together as OSKM or Yamanaka factors) [27, 28]. Since they are not derived from human embryos, their use in clinical applications faces far fewer ethical concerns than ESCs. As such, they have served as models for studying and treating various neurological disorders [29–31]. Given that iPSCs possess the same genetic information as the patients from whom they were derived, numerous studies have used patient-derived iPSCs to identify the effects of genetic background on neurological disorders, such as the role of *SNCA* multiplication in PD [32] and stress susceptibility of dopaminergic neurons with *PARKIN* mutations [33]. Patient-specific iPSC-derived neurons also serve as a drug screening platform to identify novel therapeutic targets and drug candidates for many other neurological injuries and diseases [34–38].

Moreover, because of their genetic similarity, cells and tissues derived from a patient’s iPSCs (autologous transplants) can be used for therapeutic applications without worries of immune rejection. By contrast, human leukocyte antigen (HLA)-matched allogeneic transplants, with immunocompatibility and reduced waiting times and costs for tissue generation, are also being tested. Furthermore, diverse strategies have been employed to generate HLA genome-edited hypoimmune iPSCs for even broader patient compatibility [39, 40]. Built upon robust differentiation protocols from earlier ESC studies, NCT06299033 is a Phase 1 clinical trial using allogeneic iPSC-derived NSCs to facilitate functional recovery in ischemic stroke patients (Table 1). Conversely, several PD-related clinical trials are currently underway. In Japan, clinical trial UMIN000033564, initiated in 2018 to transplant 2.5 or 5 million allogeneic iPSC-derived dopaminergic progenitors into each side of the bilateral putamen of seven patients between the ages of 50 and 70, showed significant increases in dopamine synthesis by the transplanted cells, as well as behavioral improvements among four of the six patients up to 24 months after operation [8]. This pioneering study took a similar approach to ESC trials NCT05635409 and NCT05887466 in generating dopaminergic neurons in vitro. While the same iPSC-derived product is now being tested in the U.S. (NCT06482268), multiple similar clinical trials (e.g., NCT03726814, NCT03815071, NCT03725865, NCT06145711, NCT06344026, and NCT06422208) have also been initiated recently, including several using the autologous approach for stroke and PD.

## Barriers to successful cell transplantation and integration

While the newer reports of safety and potential therapeutic benefits reaffirm the promise of stem cell–based therapies as an integral part of standard medical care for debilitating conditions such as stroke and PD, these results also suggest that further improvements to current practices are essential, even as stem cell–based therapies proceed toward regulatory approval. As noted previously [41, 42], post-mortem analyses of preclinical and clinical studies have revealed inadequate and variable survival of implanted neurons as the most likely cause of inconsistent transplantation outcomes. In both stroke and PD, the root cause of primary clinical symptoms observed in patients is the induced death of specific host neuronal populations, caused by different pathogenic mechanisms. Furthermore, while stroke and PD differ in that the former is acute and the latter is a persistent neural injury, long-term alterations occur in the tissue environment in both cases. Hence, how host neurons die as well as how their death leads to long-term and possibly permanent changes in the surrounding environment may also influence the ultimate success of cell transplantations.

Intra- and extracellular signaling pathways triggered by various stimuli, collectively known as programmed cell death (PCD), ultimately dictate neuronal survival or death. Apoptosis, the first PCD pathway identified, is essential for the normal development of the CNS and other organ systems [43, 44]. In addition, PCD also plays a critical role in normal cellular turnover and senescence, functional regulation of the immune system, control of hormone-dependent atrophy, and the cellular response to a wide range of cell stressors, including toxicological, radiological, and anoxic threats [45]. Subsequent studies have identified additional signaling pathways (e.g., necroptosis and ferroptosis) and demonstrated their contributions to disease progression.

### Programmed cell death and stroke

**Fig. 1:**
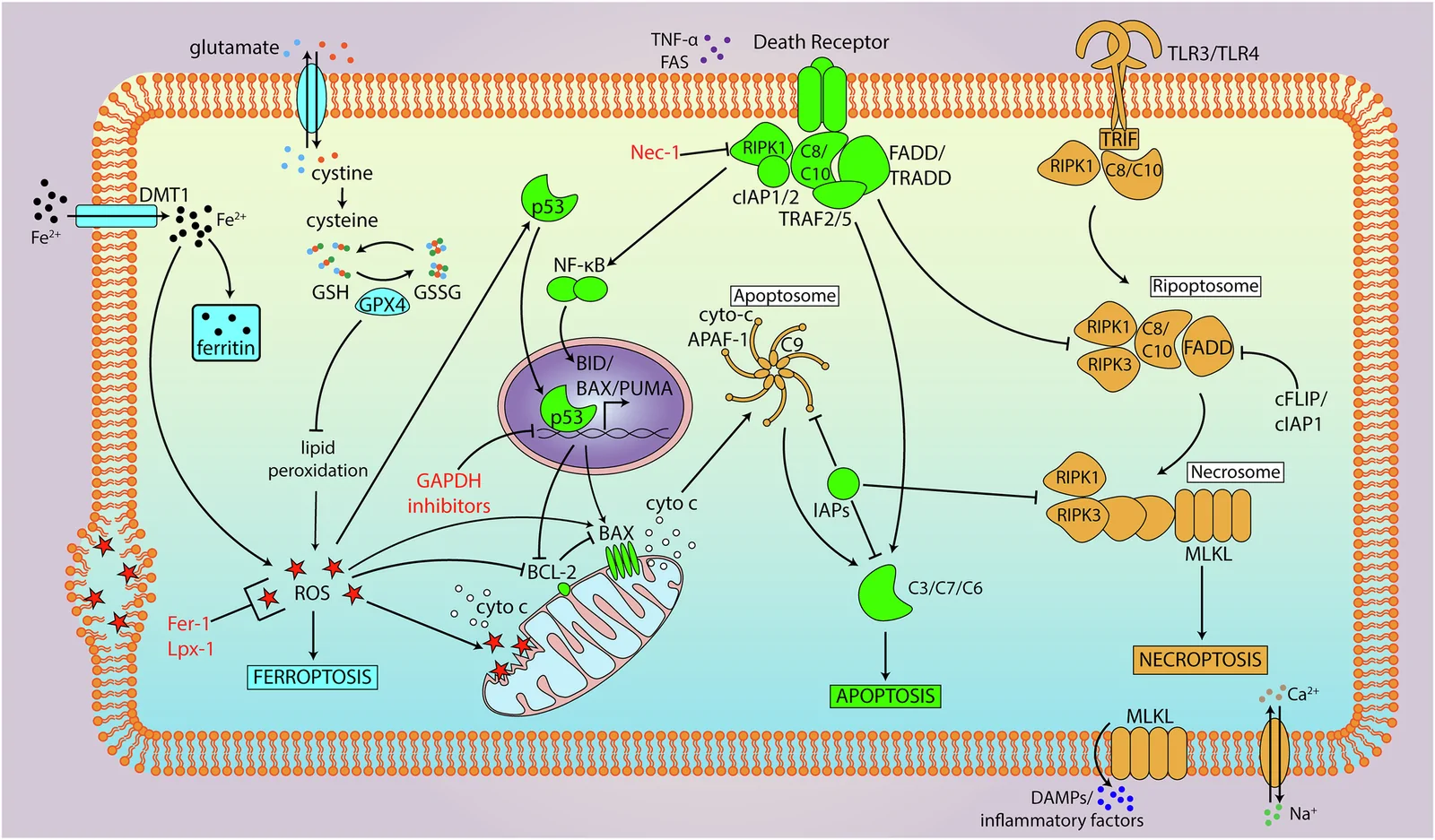
Programmed cell death pathways affecting neuronal survival and transplanted PSC-derived grafts in CNS injuries. Multiple PCD pathways—including apoptosis, necroptosis, and ferroptosis—are activated in CNS injuries and neurodegenerative disorders and can also contribute to the early loss of transplanted PSC-derived neurons. On the left, ferroptosis is triggered by iron-dependent lipid peroxidation and reduced activity of glutathione peroxidase 4 (GPX4). Inhibition by ferrostatin-1 (Fer-1), a radical scavenging antioxidant, prevents lipid ROS accumulation. In the center, interactions between pro- and anti-apoptotic BCL-2 family proteins (e.g., BCL-2, BAX, BID, PUMA, etc.) regulate apoptosis by controlling cytochrome c release, apoptosome formation, and caspase activation. On the right, necroptosis is initiated by receptor-interacting protein kinases (RIPK1/RIPK3) and mixed lineage kinase domain-like protein (MLKL), often when caspase-8 is inhibited. Death receptors and TLR3/TLR4 signaling, as well as pharmacologic modulators such as necrostatin-1, regulate these processes. Crosstalk between these pathways highlights the complexity of cell death regulation in the injured CNS and the potential benefit of multi-pronged therapeutic approaches aimed at improving neuronal survival, including the survival and integration of transplanted PSC-derived neural grafts. DAMPs, damage-associated molecular patterns; GSH, glutathione (reduced); GSSG, glutathione (oxidized); ROS, reactive oxygen species.

To understand how PCD limits the survival of PSC-based transplants, we must first understand the mechanisms of PCD that occur in stroke. During a stroke, glucose deprivation and tissue hypoxia ultimately trigger the release of the excitatory neurotransmitter glutamate in various regions of the CNS, including the neocortex [46], resulting in excessive postsynaptic activation of glutamate receptors [47]. Among these, the role of NMDA receptors, particularly those containing GluN2B [48], is crucial, as sustained activation promotes the pathological influx of extracellular Ca^2+^ and Na^+^, eventually activating downstream apoptotic and necroptotic signaling [49]. Ischemia-induced impairments in local glucose delivery and intracellular ATP production further exacerbate this process by disrupting critical ATP-dependent Na^+^/K^+^-gradient transporters as well as NKCC1- and KCC2-mediated chloride/potassium exchange [50] and SLC26A11 chloride channels [51]. In addition to promoting calpain-mediated cleavage and activation of BID, excessive Ca^2+^ influx enhances the formation of mitochondrial reactive oxygen species (ROS) in neurons through actions such as calcium/calmodulin-mediated activation of NADPH oxidases, ultimately promoting targeted bursts of ROS and tipping the balance between pro- (BAX/BAK) and anti-apoptotic (BCL-2/BCL-xL/MCL-1) BCL-2 family members toward pore formation and the promotion of mitochondrial cytochrome c release [52]. Cytosolic cytochrome c subsequently binds to Apaf-1 to initiate apoptosome formation, triggering the autocatalytic activation of caspase-9 and, ultimately, executioner caspase-3, −6, and −7, which promote the proteolytic cleavage of numerous downstream protein targets, including the nuclease caspase-activated DNase, to promote chromatin degradation and apoptotic body formation (Fig. 1, green pathway) [53]. In addition to mitochondria-mediated caspase activation (intrinsic pathway), apoptosis in neurons can also be initiated via caspase-8-mediated mechanisms (extrinsic pathway), triggered via either ligand-dependent (through cell surface death receptors such as Fas, TNFR1, and TRAIL-R1/2; death-inducing signaling complex (DISC) and complex I and IIa) or ligand-independent (ripoptosome and necrosome, also known as complex IIb and IIc, respectively) means [54].

Necroptosis represents an alternative form of ATP-dependent PCD, dependent upon the actions of receptor-interacting protein kinases (RIPKs), which become phosphorylated during ischemic brain injury [55]. Numerous studies have shown that this form of PCD predominates under conditions in which caspase-8 is either inactivated or kinetically outcompeted compared to RIPK activation. Successful RIPK1 activation promotes RIPK3 kinase phosphorylation, ultimately recruiting mixed lineage kinase domain-like protein (MLKL) for oligomerization, channel formation, and cell membrane disruption (Fig. 1, orange pathway) [56]. Necroptosis differs from apoptosis primarily due to its release of cellular content and inflammatory factors, which contribute to further brain damage [57]. Furthermore, it has been an area of intense study due to the promise of therapeutic benefits by necroptotic inhibition in rodent stroke models [58–60].

Other PCD pathways activated during ischemic stroke include iron-dependent ferroptosis, marked by mitochondria shrinkage (Fig. 1, blue pathway) [61]; autophagy, which possesses both destructive and protective roles during stroke [62]; and pro-inflammatory pyroptosis activated by cleaved gasdermin D that forms pores on the cell membrane [63]. Precisely how these pathways and their interactions contribute to neuronal cell death following stroke remains an active area of research.

### Cell death mechanisms underlying neurodegeneration in PD

As PSC-based grafts are transplanted into a degenerative environment in PD, these grafts are exposed to PCD-promoting environments. It is therefore crucial to understand the cell death mechanisms involved in PD when aiming to optimize graft survival. There are currently two pathogenic theories of PD, both of which aim to explain the loss of dopaminergic neurons in the substantia nigra pars compacta. The Lewy pathology theory proposes that the aggregation of the protein α-synuclein (Lewy body) is the cause of neuronal immunoreactivity, iron accumulation, cell toxicity, and ultimately cell death, primarily through caspase-3-dependent apoptosis, as described above [64]. By contrast, the mitochondrial dysfunction theory was established based on monogenic triggers of PD, including mutations in *PINK1*, *PARKIN*, and *CHCHD2*, all of which are associated with mitochondrial dysfunctions and, consequently, cytochrome c release [65, 66]. The two theories are not necessarily mutually exclusive, as the mitochondrial dysfunction theory could help explain the link between Lewy body accumulation and neuronal loss observed in PD patients, which is often absent in PD mouse and rat models [67–69]. Moreover, recent data suggest multiple PCD pathways may be involved in the neuronal demise observed in PD patients. Necroptosis has been observed in PD animal models, and downregulating the RIPK1/RIPK3/MLKL necroptotic pathway attenuates the loss of dopaminergic neurons substantially [69, 70]. Similarly, ferroptosis has been suggested to play a role in PD as well, with evidence showing that abnormal iron accumulation and iron-related lipid peroxidation, consequently mitochondrial membrane damage, are associated with PD progression and that ferroptosis-specific inhibitors, ferrostatin-1 (Fer-1) and liproxstatin-1 (Lpx-1), rescue neuron loss in PD models [71, 72]. Given mitochondria’s role as the nexus of apoptosis, ferroptosis, and necroptosis, more studies aimed explicitly at mitochondrial health are necessary for a potentially better understanding of PD pathology.

### Changes in the local tissue environment

While modulating cell death is crucial to limiting brain damage, regenerating neurons and neural connections are equally essential for functional recovery. Recovery after neural damage in the mammalian CNS is variable and difficult to control. Limited or dystrophic axon growth is frequently observed in lesioned areas of the CNS [73] primarily because regeneration in the adult CNS remains largely non-permissive, even under conditions where scarring is minimal [74]. Numerous intrinsic and extrinsic factors have been characterized, identifying the mechanisms preventing neural regeneration in the mature CNS. For instance, Schwann cells are crucial for clearing debris and guiding axon growth across injured areas in the PNS [75]. However, oligodendrocytes tend to limit axon growth in the CNS [76]. Furthermore, although the navigation of growth cones is crucial for establishing neural networks and axon growth, it has been shown that mature neurons in some rat models have intrinsically lost the ability to polarize and extend after neural injury [77].

## Improving PSC-based therapies

While preclinical studies have offered promising results and clinical trials have thus far demonstrated clinical safety, challenges remain in establishing these experimental therapies for reliable and widespread application. Inter-patient variability continues to plague clinical trials, with most patients experiencing therapeutic benefits while some do not. Given the invasiveness and the time and monetary costs associated with such advanced cell therapies, reproducibility must be improved. For example, the successful restoration of brain function critically depends on the ability of transplanted cells to survive, integrate, and establish appropriate neural connections within the host tissue. Next, we will discuss several crucial challenges faced by PSC-based therapies that must be addressed before broad application and examine some potential approaches.

### Cell survival and integration in patients

Although many animal studies have yielded promising results on functional improvements following cell transplantation, those findings do not necessarily translate successfully to human clinical trials for various reasons, primarily low survival and engraftment efficiency. Multiple factors, including the route of administration, disease state, and delivery method, contribute to suboptimal transplants. Next, we will examine how implanted cells and the microenvironment could be modulated to enhance post-transplantation survival and engraftment.

A primary challenge is the stability and survival of cells immediately following transplantation. In most cases, cells or, more recently, organoids in suspension are directly injected into the brain. Without further assistance and guidance, many transplanted cells fail to integrate properly at the transplantation site and die without proper trophic support. Even for those that do survive and graft, transplanted neurons must continue their differentiation trajectory to the desired cell type (i.e., mature dopaminergic neurons) and connect with their innervation targets to have therapeutic effects. While earlier studies in monkeys have shown that as few as 100,000 successfully transplanted neurons may significantly improve symptoms in human patients [78], most current clinical trials inject between 2 and 10 million cells to ensure therapeutic benefits despite the anticipated cell death following transplantation. As such, in addition to enhancing potential concerns associated with cell heterogeneity and genomic instability, this also results in an unnecessary need to produce an enormous number of cells for transplantation, ultimately driving up time and monetary costs. Furthermore, given that post-transplantation cell survival remains a stochastic process, high inter-patient variability appears unavoidable with current procedures. Thus, even nominal advances in enhancing cell survival following transplantation could dramatically alter the landscape and outlook of cell replacement therapies for CNS pathologies.

### Promoting cell survival in preclinical studies

A viable solution is to enhance the survival of transplanted cells by treating them with modulators targeting various PCD pathways described above, especially those with extensive data, including Nec-1 and caspase inhibitors. These drugs could be selectively injected along with the cell suspension during transplantation or as a pretreatment for cells before transplantation. There are currently multiple promising options, one of which is to decrease the level of TNF-α/NF-κB signaling, a conventional pro-apoptotic pathway [79]. Recently, a CRISPR-Cas9-based screen of PCD-related pathways identified p53 as a key regulator for transplanted neuron survival in vivo. Using an inducible p53 knockout cell line, cleaved caspase-3 activity was observed to accompany p53 signaling and modulated apoptosis in transplanted cells. Given the crucial nature of *TP53* as a tumor suppressor gene, its complete deletion is unsuitable for cell therapy. Instead, the authors found that antibody-based inhibition of TNF-α signaling yielded comparable improvements in engraftment as *TP53* deletion, providing a convenient and reliable approach for promoting cell survival and engraftment.

While such studies highlight how autonomous properties of transplanted cells may benefit cell survival, several recent investigations have also focused on modulating the host tissue environment. A recent study comparing differences between the injury site immediately and one week following brain injury identified recombinant progranulin as a critical factor in enhancing the engraftment of transplanted iPSC-derived neurons [80]. Reinforcing the host tissue environment as a crucial determinant of cell survival following transplantation, another study further revealed that the infiltration of inflammatory cells resulting from the injection of cells or fluid into the rat striatum led to significant cell death [81]. Validating those observations, the authors found that co-transplantation of autologous regulatory T cells with midbrain dopaminergic neurons into PD model rats dramatically reduced immediate neuronal cell death, leading to improved graft survival. Furthermore, limiting the infiltration of immune cells also reduces cytokines such as TGF-β that negatively impact neuronal progenitors commonly used in transplantations, thereby improving engraftment success. These studies demonstrate that readily applicable pre- or parallel treatments before or during transplantation can substantially improve the therapeutic efficacy of transplanted cells.

### RAS signaling as a critical modulator of post-transplantation neuronal survival and rewiring

Nonetheless, a higher survival rate does not necessarily translate to successful integration. Hence, neuronal differentiation and synaptic network reconstruction represent additional target processes for optimization. To this end, RAS signaling is a critical regulator of various cellular processes, including growth, differentiation, and survival; thus, modulating this pathway could offer significant improvements in cell replacement therapies for CNS disorders such as PD and stroke.

The molecular mechanisms of RAS, particularly in a neuronal context, have been recently reviewed [82]. RAS represents a family of cell membrane-anchored small guanosine triphosphatases (GTPases) acting as a molecular switch between an active GTP-bound and an inactive GDP-bound state [83]. Upon the binding of growth factors, hormones, or other ligands to receptor tyrosine kinases (RTKs), RAS is activated through a series of molecular events at the cell membrane [84–89] and, in turn, triggers downstream activation of MEK1/2 and ERK1/2 signaling, ultimately resulting in the induction of transcriptional programs responsible for the neuroprotective and regenerative effects of RAS [90–92].

**Fig. 2:**
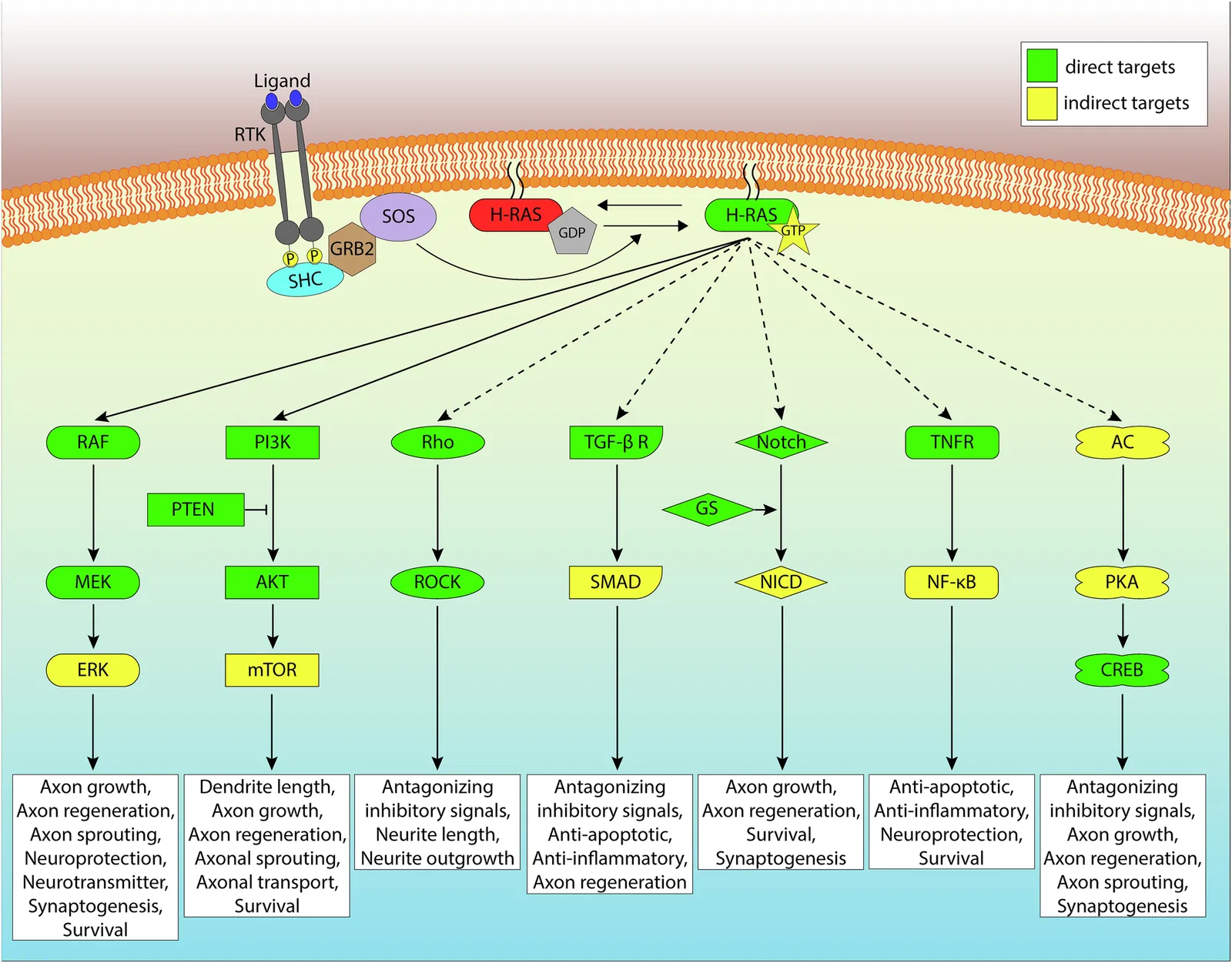
Signaling pathways promoting neuronal survival and regeneration with implications for PSC-derived neural grafts. The RAS signaling pathway functions as a central regulator of neuronal survival, neurite outgrowth, and axonal regeneration through interactions with multiple downstream signaling cascades in experimental CNS injury models. These mechanisms influence neuronal growth, differentiation, and regenerative capacity, thus providing potential molecular targets to enhance the survival, maturation, and integration of transplanted PSC-derived neurons. Proteins directly modulated to enhance neuronal survival and regeneration in the CNS in vivo are indicated in green, while those affected secondarily are indicated in yellow. Constitutive activation of RAS enhances neurite growth, survival, and neuroprotection [107]. Constitutive rapidly accelerated fibrosarcoma (RAF) activation or high-frequency repetitive transcranial magnetic stimulation-induced mitogen-activated protein kinase kinase 1/2 (MEK1/2) activation promotes corticospinal axon sprouting and recovery after spinal cord injury [159]. Phosphoinositide 3-kinase (PI3K) subunit p110δ overexpression drives CNS axon regeneration [160]. Phosphatase and tensin homolog (PTEN) antagonism enhances survival, axonal regeneration, and functional recovery post-middle cerebral artery occlusion [161]. Corticospinal tract regeneration is facilitated by overexpressing constitutive-active v-akt murine thymoma viral oncogene homolog 3 (AKT3) [162]. Dominant-negative Rho-associated protein kinase (Rho kinase) expression promotes neurite growth in severed rubrospinal tract neurons, enabling motor recovery after damage [163]. Transforming growth factor-beta type I receptor (TGF-βRI) inhibition reduces neuroinflammation and apoptosis [164]. Decreasing Notch homolog 1 (Notch1) expression via the gamma-secretase inhibitor N-[N-(3,5-difluorophenacetyl)-L-alanyl]-S-phenylglycine t-butyl ester (DAPT) improves neuronal integrity, reduces cellular edema, and alleviates mitochondrial swelling in cerebral ischemia models [165]. Tumor necrosis factor receptor 2 (TNFR2) antagonism provides neuroprotection against excitotoxicity [166]. Constitutive-active cyclic adenosine monophosphate (cAMP) response element-binding protein (CREB) expression overcomes myelin-associated axonal growth inhibition and promotes spinal axon regeneration [167].

Besides the canonical RAS/RAF/MEK/ERK pathway outlined above, active RAS engages in extensive crosstalk with multiple signaling pathways, including PI3K-AKT-mTOR [93], Rho GTPase [94], TGF-β [95], Notch [96], NF-κB [97], Hippo [98], and cAMP/PKA [99]. Due to its crucial role in these pathways, activation of RAS in neurons can promote cell survival [100], prevent apoptosis [101], increase the number of dendritic spines [102], and modulate metabolism [103], differentiation, maturation, gene expression, cytoskeletal dynamics, cell migration, etc. (Fig. 2). Additionally, a study using a conditional gain-of-function B-RAF mutant revealed that it promotes axon regeneration and outgrowth in a PI3K-mTOR-independent manner, with additive effects on axon regeneration, further demonstrating the potential of the RAS superfamily in directing neural differentiation and integration [104].

RAS signaling has been shown to exert neuroprotective effects through multiple signaling pathways in a wide range of neuronal subtypes. For example, the intracellular delivery of constitutively active H-RAS protein by trituration is sufficient to promote the survival of embryonic peripheral sensory neurons in the absence of nerve growth factor (NGF) [91]. Furthermore, constitutively active H-RAS enhances survival by suppressing the p53-mediated cell death signaling pathway in sympathetic neurons [105]. In cerebellar granule cells, RAS-MAPK signaling promotes cell survival through a dual mechanism: expression of pro-survival genes in a cAMP-responsive element-binding protein (CREB)-dependent manner and inhibition of the pro-apoptotic protein BCL-2-associated death (BAD) through phosphorylation [100]. The constitutive-active mutant H-RAS^V12^ has been shown to enhance pro-survival and regenerative signaling of RAS-related PI3K and ERK during neuronal injury, stress, and neurodegenerative disorders such as PD [106]. Specifically, SynRas mice that express H-RAS^V12^ under the control of a synapsin 1 promoter, exhibit hypertrophy of pyramidal neurons in the hippocampus and cortex, protection of motor neurons from degeneration following facial nucleus lesion, and enhanced survival of tyrosine hydroxylase (TH)-positive neurons in the substantia nigra after exposure to dopaminergic toxins [107]. Additionally, these mice show resistance to toxic hyperoxia treatment [108], indicating that neuronal RAS signaling can induce neuroprotective effects and mitigate damage from traumatic brain injury and neurodegenerative disorders such as PD. Notably, the expression of constitutively active H-RAS in post-mitotic neurons did not lead to tumor formation [107].

Among these small GTPases, H-RAS mimics neurotrophin signaling that promotes not only cell survival [109] but also axonal polarization and growth cone guidance [110]. Further validation using fluorescence resonance energy transfer-based imaging assay identified H-RAS activity as highly concentrated in the axonal growth cone of polarized neurons, further demonstrating its spatiotemporal control of neural growth [111]. Several solutions have been proposed to achieve spatial-specific H-RAS activation for applications in cell therapy. Magnetic nanoparticles (MNPs) allow a non-invasive, tissue-penetrating force to direct neurite outgrowth. One study first electroporated PC12 cells with SOS1 and H-RAS fusion protein-encoding plasmids, then introduced MNPs that selectively bind with affinity tags on the fusion proteins. By manipulating the magnetic gradient, they achieved precise spatiotemporal control of these signaling-active proteins [112]. While MNP technology requires further testing before clinical applications, the unprecedented ability to modulate intracellular signaling non-invasively at a distance provides a means to manipulate cells even after transplantation.

Based on the essential role of RAS in neurons, modulating RAS or the RAS signaling pathway offers several potential benefits for cell replacement therapies in CNS diseases [82]. Controlled activation of RAS in transplanted mature neurons may better protect them from the stressful transplantation process and the hostile environment of the diseased CNS, thereby increasing their survival rates and therapeutic efficacy. RAS signaling is associated with the growth and integration of transplanted iPSC-derived dopaminergic neurons, promoting the formation of functional neural networks [113]. RAS-mediated suppression of apoptosis through pathways such as PI3K-AKT can prevent the premature death of transplanted cells, allowing them to survive and function longer. The neuroprotective effects of RAS signaling can support both transplanted and host neurons from ongoing degenerative processes, potentially slowing disease progression and improving clinical outcomes.

Modulating the RAS signaling pathway holds great promise for improving the outcomes of cell replacement therapies in CNS diseases. RAS signaling modulation could significantly advance regenerative medicine and offer new hope for patients suffering from neurodegenerative disorders and acute neural injuries by enhancing cell survival, promoting neuronal growth, and providing neuroprotection. To leverage the full therapeutic potential of RAS, selective and temporally controlled activation in transplanted neurons or specific neuronal populations is crucial. Since the specific pharmacological activation of transplanted neurons after transplantation remains challenging, gene editing or the use of viral vectors before transplantation, enabling the controlled overexpression of constitutively active RAS under inducible or cell-type-specific promoters, appears to be a promising strategy.

### Mitochondria as a focal point of regulation for multiple PCD pathways

As outlined in the discussion thus far, through decades of research, various PCD pathways activated by acute neural injuries or during neurodegeneration, including apoptosis, necroptosis, and ferroptosis, clearly converge upon the mitochondria. Therefore, attempts have been made to genetically modify PSC-derived neurons to directly protect against mitochondrion-regulated cell death pathways and maximize transplant survival [114]. Furthermore, various strategies co-transplanting modified “helper cells” or modifying transplanted cells to secrete growth factors (e.g., GDNF and bFGF), which mediate their effects—aside from other beneficial actions—via protection against these pathways, have been demonstrated in both preclinical [115–117] and clinical studies [118]. H-RAS, notably, taps into similar signaling pathways downstream of growth factors to enhance transplant survival and functionality and thus represents a viable therapeutic target.

Targeting the PCD signaling network has been a principal research focus in the development of new stroke treatments. For example, Necrostatin-1 (Nec-1) displays neuroprotective effects by inhibiting RIPK1 and necroptosis [56] and demonstrated neuroprotection in animal models of stroke [119, 120] and AD mice [121], as well as PD cell and animal models [122–124]. Moreover, apoptosis inhibitors, including BCL-2 family modulators and those targeting effector caspases, also possess neuroprotective properties in mouse stroke [125–127] and PD models [128]. Novel strategies, including microRNA-mediated regulation of ferroportin-1 and ferroptosis, may generate a more precise modulation of the targeted PCD pathway [129–132]. Hence, because the mitochondrion is a critical convergence point for multiple PCD pathways, pre- or concurrent pharmacologic treatments before and following cell transplantation to protect against PCD may be a viable means to enhance transplanted cell survival.

Remarkably, aside from having a direct protective effect, mitochondrial metabolism is associated with and contributes significantly to the maturation of PSC-derived neurons [133, 134]. Consistently, a neuronal-like mitochondrial proteome has been shown to enhance direct astrocyte-to-neuron reprogramming [135], indicating a causative role in controlling or maintaining neuronal fate by the mitochondria and hinting at the possibility of similar genetic manipulation to improve in vitro differentiation and maturation of neurons before transplantation. Aside from metabolic changes, it is increasingly evident that mitochondrial dynamics (i.e., fission/fusion, morphology, etc.) regulate NSC properties [136, 137]. Given the availability of small molecules that can affect mitochondrial dynamics (e.g., Mdivi-1), there is a similar potential to optimize neuronal differentiation and maturation in vitro using such strategies to enhance PSC-derived neuronal production.

In recent years, partial reprogramming by intermittent expression of the Yamanaka factors has demonstrated the feasibility of cellular rejuvenation [138]. Notably, such rejuvenated cells or fully reprogrammed iPSCs exhibit properties of young cells despite being in aged animals or originating from aged donors [139–142]. Critically, full or partial reprogramming also rejuvenates mitochondrial function [138, 140], thus PSC-derived neurons and their mitochondria display favorable functional characteristics.

Altogether, overwhelming evidence highlights the mitochondrion as both a focal checkpoint and an indicator of neuronal survival and functions. Aside from taking measures to protect against cell death pathways dependent on the cellular powerhouse described above, future neural induction and quality control protocols should benefit by including steps to monitor and select neurons with optimal mitochondrial health before transplantation.

### Non-cell autonomous means to enhance neuronal survival and integration

In conjunction with other properties that may be actively modified to regulate the survival and integration of PSC-based implants, the surrounding cellular matrix has gained recognition as essential [143, 144]. Indeed, early failure of cellular attachment, which in turn stimulates processes including anoikis following transplantation, can substantially limit the success of transplantation [145]. This effect is significant enough to stimulate research toward the development of water-soluble molecules designed to protect suspended cells from anoikis by inducing integrin and heparan-sulfate-bound syndecan clustering in associated tissues, two receptor classes important for extracellular matrix (ECM)-mediated cell survival [146]. Particularly within the CNS, extracellular matrix scaffolding provides a supportive niche to promote engraftment or accelerate stem cell differentiation. Attempts to improve the survival of neural grafts and their integration by using neurotrophin-impregnated collagen hydrogels (BDNF/GDNF in the case of dopaminergic stem cell transplants) have been demonstrated to substantially improve both the survival and neural differentiation of transplanted cells in vivo [143, 147]. As expected in the case of PD, GDNF-impregnated collagen hydrogels have been used in a wide range of transplants, including the implantation of embryonic dopaminergic neurons [148]. Such neurotrophic modification has been shown to significantly increase both the long-term survival and neural integration of transplants compared to non-neurotrophin-containing hydrogels. For CNS injuries, an advantage of hydrogels is their viscoelastic properties, which can be easily modified to form a form-filling polymer while providing sufficient support for neurite outgrowth [144]. In general, it is increasingly established that the implantation of committed but functionally immature cell types typically results in better overall long-term survival and neural integration into neural networks compared to fully mature cells [149].

As for ECM composition, hyaluronic acid (HA) is one of the most frequently used hydrogel polymers due to its ubiquitous expression within the ECM [144]. Therefore, HA and HA/methylcellulose hydrogel polymers have become widely utilized for hydrogels destined for CNS applications [150, 151]. Due to their biocompatibility, relatively rapid biodegradation rate, and anti-inflammatory nature (previously shown to attenuate inflammation and inhibit glial scar formation), HA and its derivatives have been employed in a wide array of clinical CNS applications, including stroke and spinal cord injuries [144, 149, 152]. HA polymer isoforms have been shown to form a biocompatible matrix with rheological properties similar to host brain tissues, thereby supporting and promoting the survival and growth of committed cell types. Such properties could be further modified through crosslinking for stable, controlled release of neurotrophic permeants [144, 153]. Due to these properties, hyaluronic scaffolds have become increasingly utilized in live-cell tissue printing applications, where their matrix properties enable detailed cellular alignment within 3D printing applications, such as the spinal cord [154]. To further enhance the neuro-integrative properties of HA hydrogels, additional components, including laminins, perlecans, and chondroitin sulfate proteoglycans such as tenascins, are being incorporated into matrices to promote local β1 integrin binding. Such interaction has been demonstrated to regulate the formation and fine-tuning of neural circuitry during development [155, 156]. Consistent with this, the loss of β1-dependent or reelin (via binding to β1 integrin-containing receptors) signaling impedes synapse formation, thus reducing the number of synaptic spines in the hippocampus [157]. Therefore, recreating the permissive environment for neural integration during neurodevelopment should allow transplanted cells to more readily adapt and mesh with existing neural networks.

## Conclusion

In the past few decades, we have witnessed not only significant progress in understanding CNS injuries and disease but also a staggering boom in regenerative medicine. Research on new PSC-based therapeutic approaches highlights optimism for treating neurological pathologies—especially given their capabilities in tissue replacement and neural rewiring—challenging tasks for conventional drug therapies. However, as we embrace the era of mass investment and expansion in regenerative medicine, foreseeable challenges persist before research advances translate into widely available therapies. Many advanced therapies in clinical trials struggle to benefit from preclinical improvements. The complicated nature of the human CNS continues to hinder clinical translation. Changes to different aspects of PSC-based therapies are necessary to revolutionize them for broad applicability (Box 1). We should also remind ourselves that it has been merely two decades since the discovery of iPSCs. While most PSC-based therapies are still in early development, we shall remain optimistic and confident about the future of these treatments. The evolution of stem cells and related technologies for regenerative medicine represents a relatively new frontier in biomedical science, which requires continued investment and interdisciplinary collaboration. Based on recent advances in both the basic and the clinical research field, we propose three approaches to overcome challenges in achieving successful and reproducible PSC-based therapies: (1) enhancing transplanted cell survival through pharmacological inhibition of PCD pathways or genome editing, (2) promoting neuronal differentiation and maturation of transplanted cells by inducible RAS signaling activation and mitochondrial rejuvenation, and (3) optimizing neuronal integration and rewiring using extracellular matrix scaffolds and molecular guidance cues. As these strategies operate at different mechanistic levels, they could be combined in a modular manner to maximize graft survival and functional integration.

While clinical iPSC-based transplantation efforts are currently most advanced in PD and stroke, similar challenges in graft survival and integration are expected to apply to other neurodegenerative disorders such as AD, amyotrophic lateral sclerosis, and Huntington’s disease. At present, most iPSC-based approaches for these conditions remain preclinical or in vitro; however, the strategies discussed here may inform future translational efforts across a broad range of CNS disorders [158].

As the scientific community continues to push the boundaries of what is possible, the next generation of stem cell therapy stands poised to bring transformative changes to our approach to treating CNS pathologies, thus offering renewed hope for patients and their families.

Box 1 Strategies to enhance the survival and integration of transplanted PSC-based products *Pre-transplantation*

- Transient overexpression or knockdown of proteins to promote lineage specification or eliminate non-target cells
- Pre-conditioning cells for hostile microenvironments (e.g., hypoxia, growth factors, etc.)
- Cell type-specific purification
- Viability and vitality checks (e.g., functional assays)

*Transplantation*

- Novel transplantation apparatus to enhance graft survival and stability
- Biocompatible materials to stabilize transplanted cells for grafting
- Embedded growth factors or axon guidance cues to stimulate survival and neurite outgrowth

*Post-transplantation*

- Anti-apoptotic modulation (genetic or pharmacologic means)
- Magnetic nanoparticle-mediated axon guidance

## References

[1] GBD 2021 Nervous System Disorders Collaborators, Steinmetz JD, Seeher KM, Schiess N, Nichols E, Cao B, et al. Global, regional, and national burden of disorders affecting the nervous system, 1990–2021: a systematic analysis for the Global Burden of Disease Study 2021. Lancet Neurol. 2024;23:344–81. 10.1016/S1474-4422(24)00038-3 PMC10949203 38493795

[2] GBD 2019 Stroke Collaborators, Feigin VL, Stark BA, Johnson CO, Roth GA, Bisignano C, et al. Global, regional, and national burden of stroke and its risk factors, 1990–2019: a systematic analysis for the Global Burden of Disease Study 2019. Lancet Neurol. 2021;20:795–820. 10.1016/S1474-4422(21)00252-0 PMC8443449 34487721

[3] Feigin VL, Brainin M, Norrving B, Martins S, Sacco RL, Hacke W, et al. World Stroke Organization (WSO): Global Stroke Fact Sheet 2022. Int J Stroke. 2021;17:18–29. 10.1177/17474930211065917 34986727

[4] Leng F, Edison P. Neuroinflammation and microglial activation in Alzheimer disease: where do we go from here? Nat Rev Neurol. 2021;17:157–72. 10.1038/s41582-020-00435-y 33318676

[5] Kikuchi T, Morizane A, Doi D, Magotani H, Onoe H, Hayashi T, et al. Human iPS cell-derived dopaminergic neurons function in a primate Parkinson’s disease model. Nature. 2017;548:592–6. 10.1038/nature23664 28858313

[6] Doi D, Magotani H, Kikuchi T, Ikeda M, Hiramatsu S, Yoshida K, et al. Pre-clinical study of induced pluripotent stem cell-derived dopaminergic progenitor cells for Parkinson’s disease. Nat Commun. 2020;11:3369. 10.1038/s41467-020-17165-w PMC7338530 32632153

[7] Tabar V, Sarva H, Lozano AM, Fasano A, Kalia SK, Yu KKH, et al. Phase I trial of hES cell-derived dopaminergic neurons for Parkinson’s disease. Nature. 2025;641:978–83. 10.1038/s41586-025-08845-y PMC12095069 40240592

[8] Sawamoto N, Doi D, Nakanishi E, Sawamura M, Kikuchi T, Yamakado H, et al. Phase I/II trial of iPS-cell-derived dopaminergic cells for Parkinson’s disease. Nature. 2025;641:971–7. 10.1038/s41586-025-08700-0 PMC12095070 40240591

[9] Olanow CW, Goetz CG, Kordower JH, Stoessl AJ, Sossi V, Brin MF, et al. A double-blind controlled trial of bilateral fetal nigral transplantation in Parkinson’s disease. Ann Neurol. 2003;54:403–14. 10.1002/ana.10720 12953276

[10] Spencer DD, Robbins RJ, Naftolin F, Marek KL, Vollmer T, Leranth C, et al. Unilateral transplantation of human fetal mesencephalic tissue into the caudate nucleus of patients with Parkinson’s disease. N Engl J Med. 1992;327:1541–8. 10.1056/NEJM199211263272201 1435880

[11] Liu J, Huang H-Y. How to improve the survival of the fetal ventral mesencephalic cell transplanted in Parkinson’s disease? Neurosci Bull. 2007;23:377–82. 10.1007/s12264-007-0056-4 PMC5550653 18064069

[12] Barker RA, Farrell K, Guzman NV, He X, Lazic SE, Moore S, et al. Designing stem-cell-based dopamine cell replacement trials for Parkinson’s disease. Nat Med. 2019;25:1045–53. 10.1038/s41591-019-0507-2 31263283

[13] Zhang Y, Pak C, Han Y, Ahlenius H, Zhang Z, Chanda S, et al. Rapid single-step induction of functional neurons from human pluripotent stem cells. Neuron. 2013;78:785–98. 10.1016/j.neuron.2013.05.029 PMC3751803 23764284

[14] Huang C, Su T, Xue Y, Cheng C, Lay FD, McKee RA, et al. Cbx3 maintains lineage specificity during neural differentiation. Genes Dev. 2017;31:241–6. 10.1101/gad.292169.116 PMC5358721 28270516

[15] Xu H, Fan X, Wu X, Tang J, Yang H. Neural precursor cells differentiated from mouse embryonic stem cells relieve symptomatic motor behavior in a rat model of Parkinson’s disease. Biochem Biophys Res Commun. 2004;326:115–22. 10.1016/j.bbrc.2004.10.210 15567160

[16] Pera MF, Andrade J, Houssami S, Reubinoff B, Trounson A, Stanley EG, et al. Regulation of human embryonic stem cell differentiation by BMP-2 and its antagonist noggin. J Cell Sci. 2004;117:1269–80. 10.1242/jcs.00970 14996946

[17] Chambers SM, Fasano CA, Papapetrou EP, Tomishima M, Sadelain M, Studer L. Highly efficient neural conversion of human ES and iPS cells by dual inhibition of SMAD signaling. Nat Biotechnol. 2009;27:275–80. 10.1038/nbt.1529 PMC2756723 19252484

[18] Björklund LM, Sánchez-Pernaute R, Chung S, Andersson T, Chen IYC, McNaught KSTP, et al. Embryonic stem cells develop into functional dopaminergic neurons after transplantation in a Parkinson rat model. Proc Natl Acad Sci USA. 2002;99:2344–9. 10.1073/pnas.022438099 PMC122367 11782534

[19] Ben-Hur T, Idelson M, Khaner H, Pera M, Reinhartz E, Itzik A, et al. Transplantation of human embryonic stem cell–derived neural progenitors improves behavioral deficit in Parkinsonian rats. Stem Cells. 2004;22:1246–55. 10.1634/stemcells.2004-0094 15579643

[20] Takagi Y, Takahashi J, Saiki H, Morizane A, Hayashi T, Kishi Y, et al. Dopaminergic neurons generated from monkey embryonic stem cells function in a Parkinson primate model. J Clin Investig. 2005;115:102–9. 10.1172/JCI21137 PMC539189 15630449

[21] Wang Y-K, Zhu W-W, Wu M-H, Wu Y-H, Liu Z-X, Liang L-M, et al. Human clinical-grade parthenogenetic ESC-derived dopaminergic neurons recover locomotive defects of nonhuman primate models of Parkinson’s disease. Stem Cell Rep. 2018;11:171–82. 10.1016/j.stemcr.2018.05.010 PMC6067059 29910127

[22] Emborg ME, Zhang Z, Joers V, Brunner K, Bondarenko V, Ohshima S, et al. Intracerebral transplantation of differentiated human embryonic stem cells to hemiparkinsonian monkeys. Cell Transplant. 2013;22:831–8. 10.3727/096368912X647144 PMC3728183 23594934

[23] Ikeda R, Kurokawa MS, Chiba S, Yoshikawa H, Ide M, Tadokoro M, et al. Transplantation of neural cells derived from retinoic acid-treated cynomolgus monkey embryonic stem cells successfully improved motor function of hemiplegic mice with experimental brain injury. Neurobiol Dis. 2005;20:38–48. 10.1016/j.nbd.2005.01.031 16137565

[24] Steinberg G, Bet A, Williams J, McDonald K, Diaz RJ, Samos CH, et al. Abstract 26: First-in-human phase 1/2a study of intracerebral transplantation using embryonic-derived neural stem cells (NR1) for chronic ischemic stroke (NCT04631406): 12 months outcomes. Stroke. 2025;56:A26–A26.

[25] Park S, Park CW, Eom JH, Jo M-Y, Hur H-J, Choi SK, et al. Preclinical and dose-ranging assessment of hESC-derived dopaminergic progenitors for a clinical trial on Parkinson’s disease. Cell Stem Cell. 2024;31:25–38. e8. 10.1016/j.stem.2023.11.009 38086390

[26] Kirkeby A, Nelander J, Hoban DB, Rogelius N, Bjartmarz H, NNCT RD, et al. Preclinical quality, safety, and efficacy of a human embryonic stem cell-derived product for the treatment of Parkinson’s disease, STEM-PD. Cell Stem Cell. 2023;30:1299–314. e9. 10.1016/j.stem.2023.08.014 37802036

[27] Takahashi K, Yamanaka S. Induction of pluripotent stem cells from mouse embryonic and adult fibroblast cultures by defined factors. Cell. 2006;126:663–76. 10.1016/j.cell.2006.07.024 16904174

[28] Takahashi K, Tanabe K, Ohnuki M, Narita M, Ichisaka T, Tomoda K, et al. Induction of pluripotent stem cells from adult human fibroblasts by defined factors. Cell. 2007;131:861–72. 10.1016/j.cell.2007.11.019 18035408

[29] Liu Q, Waltz S, Woodruff G, Ouyang J, Israel MA, Herrera C, et al. Effect of potent γ-secretase modulator in human neurons derived from multiple presenilin 1–induced pluripotent stem cell mutant carriers. JAMA Neurol. 2014;71:1481–9. 10.1001/jamaneurol.2014.2482 PMC4374637 25285942

[30] Laperle AH, Sances S, Yucer N, Dardov VJ, Garcia VJ, Ho R, et al. iPSC modeling of young-onset Parkinson’s disease reveals a molecular signature of disease and novel therapeutic candidates. Nat Med. 2020;26:289–99. 10.1038/s41591-019-0739-1 31988461

[31] Okano H, Morimoto S. iPSC-based disease modeling and drug discovery in cardinal neurodegenerative disorders. Cell Stem Cell. 2022;29:189–208. 10.1016/j.stem.2022.01.007 35120619

[32] Prots I, Grosch J, Brazdis R-M, Simmnacher K, Veber V, Havlicek S, et al. α-Synuclein oligomers induce early axonal dysfunction in human iPSC-based models of synucleinopathies. Proc Natl Acad Sci USA. 2018;115:7813–8. 10.1073/pnas.1713129115 PMC6065020 29991596

[33] Chung SY, Kishinevsky S, Mazzulli JR, Graziotto J, Mrejeru A, Mosharov EV, et al. Parkin and PINK1 patient iPSC-derived midbrain dopamine neurons exhibit mitochondrial dysfunction and α-synuclein accumulation. Stem Cell Rep. 2016;7:664–77. 10.1016/j.stemcr.2016.08.012 PMC5063469 27641647

[34] Li W-Y, Zhu Q-B, Jin L-Y, Yang Y, Xu X-Y, Hu X-Y. Exosomes derived from human induced pluripotent stem cell-derived neural progenitor cells protect neuronal function under ischemic conditions. Neural Regen Res. 2021;16:2064–70. 10.4103/1673-5374.308665 PMC8343330 33642395

[35] Tabata Y, Imaizumi Y, Sugawara M, Andoh-Noda T, Banno S, Chai M, et al. T-type calcium channels determine the vulnerability of dopaminergic neurons to mitochondrial stress in familial Parkinson disease. Stem Cell Rep. 2018;11:1171–84. 10.1016/j.stemcr.2018.09.006 PMC6234903 30344006

[36] Egawa N, Kitaoka S, Tsukita K, Naitoh M, Takahashi K, Yamamoto T, et al. Drug screening for ALS using patient-specific induced pluripotent stem cells. Sci Transl Med. 2012;4:145ra104. 10.1126/scitranslmed.3004052 22855461

[37] Imamura K, Izumi Y, Watanabe A, Tsukita K, Woltjen K, Yamamoto T et al. The Src/c-Abl pathway is a potential therapeutic target in amyotrophic lateral sclerosis. Sci Transl Med. 2017;9. 10.1126/scitranslmed.aaf3962. 28539470

[38] Kondo T, Imamura K, Funayama M, Tsukita K, Miyake M, Ohta A, et al. iPSC-based compound screening and in vitro trials identify a synergistic anti-amyloid β combination for Alzheimer’s disease. Cell Rep. 2017;21:2304–12. 10.1016/j.celrep.2017.10.109 29166618

[39] Xu H, Wang B, Ono M, Kagita A, Fujii K, Sasakawa N, et al. Targeted disruption of HLA genes via CRISPR-Cas9 generates iPSCs with enhanced immune compatibility. Cell Stem Cell. 2019;24:566–78. e7. 10.1016/j.stem.2019.02.005 30853558

[40] Gravina A, Tediashvili G, Zheng Y, Iwabuchi KA, Peyrot SM, Roodsari SZ, et al. Synthetic immune checkpoint engagers protect HLA-deficient iPSCs and derivatives from innate immune cell cytotoxicity. Cell Stem Cell. 2023;30:1538–48. e4. 10.1016/j.stem.2023.10.003 37922880

[41] Brundin P, Karlsson J, Emgård M, Schierle GSK, Hansson O, Petersén Å, et al. Improving the survival of grafted dopaminergic neurons: a review over current approaches. Cell Transplant. 2000;9:179–95. 10.1177/096368970000900205 10811392

[42] Park T-Y, Jeon J, Cha Y, Kim K-S. Past, present, and future of cell replacement therapy for parkinson’s disease: a novel emphasis on host immune responses. Cell Res. 2024;34:479–92. 10.1038/s41422-024-00971-y PMC11217403 38777859

[43] Yamaguchi Y, Miura M. Programmed cell death in neurodevelopment. Dev Cell. 2015;32:478–90. 10.1016/j.devcel.2015.01.019 25710534

[44] Nguyen TTM, Gillet G, Popgeorgiev N. Caspases in the developing central nervous system: apoptosis and beyond. Front Cell Dev Biol. 2021;9:702404. 10.3389/fcell.2021.702404 PMC8322698 34336853

[45] Singh R, Letai A, Sarosiek K. Regulation of apoptosis in health and disease: the balancing act of BCL-2 family proteins. Nat Rev Mol Cell Biol. 2019;20:175–93. 10.1038/s41580-018-0089-8 PMC7325303 30655609

[46] Wu QJ, Tymianski M. Targeting NMDA receptors in stroke: new hope in neuroprotection. Mol Brain. 2018;11:15. 10.1186/s13041-018-0357-8 PMC5851248 29534733

[47] Wang Z, Zhang X, Zhang G, Zheng YJ, Zhao A, Jiang X, et al. Astrocyte modulation in cerebral ischemia-reperfusion injury: A promising therapeutic strategy. Exp Neurol. 2024;378:114814. 10.1016/j.expneurol.2024.114814 38762094

[48] Liu Y, Wong TP, Aarts M, Rooyakkers A, Liu L, Lai TW, et al. NMDA receptor subunits have differential roles in mediating excitotoxic neuronal death both in vitro and in vivo. J Neurosci. 2007;27:2846–57. 10.1523/JNEUROSCI.0116-07.2007 PMC6672582 17360906

[49] Li V, Wang YT. Molecular mechanisms of NMDA receptor-mediated excitotoxicity: implications for neuroprotective therapeutics for stroke. Neural Regen Res. 2016;11:1752–3. 10.4103/1673-5374.194713 PMC5204222 28123410

[50] Loh KY, Wang Z, Liao P. Oncotic cell death in stroke. Rev Physiol Biochem Pharmacol. 2018;176:37–64. 10.1007/112_2018_13 30515590

[51] Wei S, Chen B, Low SW, Poore CP, Gao Y, Nilius B, et al. SLC26A11 inhibition reduces oncotic neuronal death and attenuates stroke reperfusion injury. Mol Neurobiol. 2023;60:5931–43. 10.1007/s12035-023-03453-1 PMC10471688 37380823

[52] Czabotar PE, Garcia-Saez AJ. Mechanisms of BCL-2 family proteins in mitochondrial apoptosis. Nat Rev Mol Cell Biol. 2023;24:732–48. 10.1038/s41580-023-00629-4 37438560

[53] Sahoo G, Samal D, Khandayataray P, Murthy MK. A review on caspases: key regulators of biological activities and apoptosis. Mol Neurobiol. 2023;60:5805–37. 10.1007/s12035-023-03433-5 37349620

[54] Tummers B, Green DR. Caspase-8: regulating life and death. Immunol Rev. 2017;277:76–89. 10.1111/imr.12541 PMC5417704 28462525

[55] Mifflin L, Ofengeim D, Yuan J. Receptor-interacting protein kinase 1 (RIPK1) as a therapeutic target. Nat Rev Drug Discov. 2020;19:553–71. 10.1038/s41573-020-0071-y PMC7362612 32669658

[56] Degterev A, Huang Z, Boyce M, Li Y, Jagtap P, Mizushima N, et al. Chemical inhibitor of nonapoptotic cell death with therapeutic potential for ischemic brain injury. Nat Chem Biol. 2005;1:112–9. 10.1038/nchembio711 16408008

[57] He S, Wang L, Miao L, Wang T, Du F, Zhao L, et al. Receptor interacting protein kinase-3 determines cellular necrotic response to TNF-α. Cell. 2009;137:1100–11. 10.1016/j.cell.2009.05.021 19524512

[58] Lule S, Wu L, Sarro-Schwartz A, Edmiston WJ, Izzy S, Songtachalert T, et al. Cell-specific activation of RIPK1 and MLKL after intracerebral hemorrhage in mice. J Cereb Blood Flow Metab. 2020;41:1623–33. 10.1177/0271678X20973609 PMC8221773 33210566

[59] Chevin M, Chabrier S, Allard M-J, Sébire G. Necroptosis blockade potentiates the neuroprotective effect of hypothermia in neonatal hypoxic-ischemic encephalopathy. Biomedicines. 2022;10:2913. 10.3390/biomedicines10112913 PMC9687213 36428481

[60] Soeandy CD, Elia AJ, Cao Y, Rodgers C, Huang S, Elia AC, et al. Necroptotic–apoptotic regulation in an endothelin-1 model of cerebral ischemia. Cell Mol Neurobiol. 2021;41:1727–42. 10.1007/s10571-020-00942-y PMC11444014 32844322

[61] Hanson LR, Roeytenberg A, Martinez PM, Coppes VG, Sweet DC, Rao RJ, et al. Intranasal deferoxamine provides increased brain exposure and significant protection in rat ischemic stroke. J Pharmacol Exp Ther. 2009;330:679–86. 10.1124/jpet.108.149807 PMC2729791 19509317

[62] Liu S, Yao S, Yang H, Liu S, Wang Y. Autophagy: regulator of cell death. Cell Death Dis. 2023;14:648. 10.1038/s41419-023-06154-8 PMC10551038 37794028

[63] Hu R, Liang J, Ding L, Zhang W, Wang Y, Zhang Y, et al. Gasdermin D inhibition ameliorates neutrophil mediated brain damage in acute ischemic stroke. Cell Death Discov. 2023;9:50. 10.1038/s41420-023-01349-6 PMC9908898 36755018

[64] Taylor J-P, McKeith IG, Burn DJ, Boeve BF, Weintraub D, Bamford C, et al. New evidence on the management of Lewy body dementia. Lancet Neurol. 2020;19:157–69. 10.1016/S1474-4422(19)30153-X PMC7017451 31519472

[65] Langston JW, Ballard P, Tetrud JW, Irwin I. Chronic parkinsonism in humans due to a product of meperidine-analog synthesis. Science. 1983;219:979–80. 10.1126/science.6823561 6823561

[66] Funayama M, Ohe K, Amo T, Furuya N, Yamaguchi J, Saiki S, et al. CHCHD2 mutations in autosomal dominant late-onset Parkinson’s disease: a genome-wide linkage and sequencing study. Lancet Neurol. 2015;14:274–82. 10.1016/S1474-4422(14)70266-2 25662902

[67] Perier C, Bové J, Wu D-C, Dehay B, Choi D-K, Jackson-Lewis V, et al. Two molecular pathways initiate mitochondria-dependent dopaminergic neurodegeneration in experimental Parkinson’s disease. Proc Natl Acad Sci USA. 2007;104:8161–6. 10.1073/pnas.0609874104 PMC1876588 17483459

[68] Palacino JJ, Sagi D, Goldberg MS, Krauss S, Motz C, Wacker M, et al. Mitochondrial dysfunction and oxidative damage in parkin-deficient mice. J Biol Chem. 2004;279:18614–22. 10.1074/jbc.M401135200 14985362

[69] Lin Q-S, Chen P, Wang W-X, Lin C-C, Zhou Y, Yu L-H, et al. RIP1/RIP3/MLKL mediates dopaminergic neuron necroptosis in a mouse model of Parkinson disease. Lab Investig. 2020;100:503–11. 10.1038/s41374-019-0319-5 31506635

[70] Kartik S, Pal R, Chaudhary MJ, Tiwari PC, Nath R, Kumar M. Anti-oxidative and anti-neuroinflammatory role of Necrostatin-1s and docosahexaenoic acid in RIP-1-mediated neurotoxicity in MPTP-induced Parkinson’s disease model. Fundam Clin Pharmacol. 2023;37:794–806. 10.1111/fcp.12881 36807936

[71] Van BD, Gouel F, Jonneaux A, Timmerman K, Gelé P, Pétrault M, et al. Ferroptosis, a newly characterized form of cell death in Parkinson’s disease that is regulated by PKC. Neurobiol Dis. 2016;94:169–78. 10.1016/j.nbd.2016.05.011 27189756

[72] Zhang B, Chen K, Dai Y, Luo X, Xiong Z, Zhang W, et al. Human α-synuclein aggregation activates ferroptosis leading to parvalbumin interneuron degeneration and motor learning impairment. Commun Biol. 2024;7:1227. 10.1038/s42003-024-06896-x PMC11443099 39349708

[73] Varadarajan SG, Hunyara JL, Hamilton NR, Kolodkin AL, Huberman AD. Central nervous system regeneration. Cell. 2022;185:77–94. 10.1016/j.cell.2021.10.029 PMC10896592 34995518

[74] Canty AJ, Huang L, Jackson JS, Little GE, Knott G, Maco B, et al. In-vivo single neuron axotomy triggers axon regeneration to restore synaptic density in specific cortical circuits. Nat Commun. 2013;4:2038. 10.1038/ncomms3038 23799397

[75] Corfas G, Velardez MO, Ko C-P, Ratner N, Peles E. Mechanisms and roles of Axon-Schwann cell interactions. J Neurosci. 2004;24:9250–60. 10.1523/JNEUROSCI.3649-04.2004 PMC6730082 15496660

[76] Iwashita Y, Blakemore WF. Areas of demyelination do not attract significant numbers of Schwann cells transplanted into normal white matter. Glia. 2000;31:232–40. 10.1002/1098-1136(200009)31:3<232::aid-glia40>3.0.co;2-8 10941149

[77] Davies SJA, Fitch MT, Memberg SP, Hall AK, Raisman G, Silver J. Regeneration of adult axons in white matter tracts of the central nervous system. Nature. 1997;390:680–3. 10.1038/37776 9414159

[78] Kikuchi T, Morizane A, Doi D, Onoe H, Hayashi T, Kawasaki T, et al. Survival of human induced pluripotent stem cell–derived midbrain dopaminergic neurons in the brain of a primate model of Parkinson’s disease. J Parks Dis. 2011;1:395–412. 10.3233/JPD-2011-11070 23933658

[79] Kim TW, Koo SY, Riessland M, Chaudhry F, Kolisnyk B, Cho HS et al. TNF-NF-κB-p53 axis restricts in vivo survival of hPSC-derived dopamine neurons. Cell. 2024. 10.1016/j.cell.2024.05.030. PMC11641762 38866017

[80] Yamagami K, Samata B, Doi D, Tsuchimochi R, Kikuchi T, Amimoto N, et al. Progranulin enhances the engraftment of transplanted human iPS cell-derived cerebral neurons. Stem Cells Transl Med. 2024;13:1113–28. 10.1093/stcltm/szae066 PMC11555480 39340829

[81] Park T-Y, Jeon J, Lee N, Kim J, Song B, Kim J-H, et al. Co-transplantation of autologous Treg cells in a cell therapy for Parkinson’s disease. Nature. 2023;619:606–15. 10.1038/s41586-023-06300-4 PMC12012854 37438521

[82] Schöneborn H, Raudzus F, Coppey M, Neumann S, Heumann R. Perspectives of RAS and RHEB GTPase signaling pathways in regenerating brain neurons. Int J Mol Sci. 2018;19:4052. 10.3390/ijms19124052 PMC6321366 30558189

[83] Vetter IR, Wittinghofer A. The guanine nucleotide-binding switch in three dimensions. Science. 2001;294:1299–304. 10.1126/science.1062023 11701921

[84] Tomuleasa C, Tigu A-B, Munteanu R, Moldovan C-S, Kegyes D, Onaciu A, et al. Therapeutic advances of targeting receptor tyrosine kinases in cancer. Signal Transduct Target Ther. 2024;9:201. 10.1038/s41392-024-01899-w PMC11323831 39138146

[85] Lowenstein EJ, Daly RJ, Batzer AG, Li W, Margolis B, Lammers R, et al. The SH2 and SH3 domain-containing protein GRB2 links receptor tyrosine kinases to ras signaling. Cell. 1992;70:431–42. 10.1016/0092-8674(92)90167-b 1322798

[86] Gale NW, Kaplan S, Lowenstein EJ, Schlessinger J, Bar-Sagi D. Grb2 mediates the EGF-dependent activation of guanine nucleotide exchange on Ras. Nature. 1993;363:88–92. 10.1038/363088a0 8386805

[87] Buday L, Downward J. Epidermal growth factor regulates p21ras through the formation of a complex of receptor, Grb2 adapter protein, and Sos nucleotide exchange factor. Cell. 1993;73:611–20. 10.1016/0092-8674(93)90146-h 8490966

[88] Tran TH, Chan AH, Young LC, Bindu L, Neale C, Messing S, et al. KRAS interaction with RAF1 RAS-binding domain and cysteine-rich domain provides insights into RAS-mediated RAF activation. Nat Commun. 2021;12:1176. 10.1038/s41467-021-21422-x PMC7895934 33608534

[89] Dougherty MK, Müller J, Ritt DA, Zhou M, Zhou XZ, Copeland TD, et al. Regulation of Raf-1 by Direct Feedback Phosphorylation. Mol Cell. 2005;17:215–24. 10.1016/j.molcel.2004.11.055 15664191

[90] Bar-Sagi D, Feramisco JR. Microinjection of the ras oncogene protein into PC12 cells induces morphological differentiation. Cell. 1985;42:841–8. 10.1016/0092-8674(85)90280-6 2996779

[91] Borasio GD, John J, Wittinghofer A, Barde Y-A, Sendtner M, Heumann R. ras p21 protein promotes survival and fiber outgrowth of cultured embryonic neurons. Neuron. 1989;2:1087–96. 10.1016/0896-6273(89)90233-x 2696501

[92] Mebratu Y, Tesfaigzi Y. How ERK1/2 activation controls cell proliferation and cell death: Is subcellular localization the answer? Cell Cycle. 2009;8:1168–75. 10.4161/cc.8.8.8147 PMC2728430 19282669

[93] Mendoza MC, Er EE, Blenis J. The Ras-ERK and PI3K-mTOR pathways: cross-talk and compensation. Trends Biochem Sci. 2011;36:320–8. 10.1016/j.tibs.2011.03.006 PMC3112285 21531565

[94] Jaiswal M, Dvorsky R, Amin E, Risse SL, Fansa EK, Zhang S-C, et al. Functional cross-talk between ras and rho pathways: a Ras-specific GTPase-activating protein (p120RasGAP) competitively inhibits the RhoGAP activity of deleted in liver cancer (DLC) tumor suppressor by masking the catalytic arginine finger. J Biol Chem. 2014;289:6839–49. 10.1074/jbc.M113.527655 PMC3945346 24443565

[95] Liu S, Iaria J, Simpson RJ, Zhu H-J. Ras enhances TGF-β signaling by decreasing cellular protein levels of its type II receptor negative regulator SPSB1. Cell Commun Signal CCS. 2018;16:10. 10.1186/s12964-018-0223-4 PMC5850916 29534718

[96] Carmena A, Buff E, Halfon MS, Gisselbrecht S, Jiménez F, Baylies MK, et al. Reciprocal regulatory interactions between the Notch and Ras signaling pathways in the drosophila embryonic mesoderm. Dev Biol. 2002;244:226–42. 10.1006/dbio.2002.0606 11944933

[97] Tago K, Funakoshi-Tago M, Ohta S, Kawata H, Saitoh H, Horie H, et al. Oncogenic Ras mutant causes the hyperactivation of NF-κB via acceleration of its transcriptional activation. Mol Oncol. 2019;13:2493–510. 10.1002/1878-0261.12580 PMC6822247 31580526

[98] Pascual J, Jacobs J, Sansores-Garcia L, Natarajan M, Zeitlinger J, Aerts S, et al. Hippo reprograms the transcriptional response to ras signaling. Dev Cell. 2017;42:667–80. e4. 10.1016/j.devcel.2017.08.013 28950103

[99] Yu Q, Gong X, Tong Y, Wang M, Duan K, Zhang X, et al. Phosphorylation of Jhd2 by the Ras-cAMP-PKA(Tpk2) pathway regulates histone modifications and autophagy. Nat Commun. 2022;13:5675. 10.1038/s41467-022-33423-5 PMC9515143 36167807

[100] Bonni A, Brunet A, West AE, Datta SR, Takasu MA, Greenberg ME. Cell survival promoted by the Ras-MAPK signaling pathway by transcription-dependent and -independent mechanisms. Science. 1999;286:1358–62. 10.1126/science.286.5443.1358 10558990

[101] Serdar M, Herz J, Kempe K, Winterhager E, Jastrow H, Heumann R, et al. Protection of oligodendrocytes through neuronal overexpression of the small GTPase Ras in hyperoxia-induced neonatal brain injury. Front Neurol. 2018;9:175. 10.3389/fneur.2018.00175 PMC5871665 29619004

[102] Krüssel S, Deb I, Son S, Ewall G, Chang M, Lee H-K et al. Exuberant de novo dendritic spine growth in mature neurons. bioRxiv. 2023; 2023.07.21.550095. 10.1016/j.isci.2024.110535 PMC11365382 39220408

[103] Botero V, Tomchik SM. Unraveling neuronal and metabolic alterations in neurofibromatosis type 1. J Neurodev Disord. 2024;16:49. 10.1186/s11689-024-09565-6 PMC11365184 39217323

[104] O’Donovan KJ, Ma K, Guo H, Wang C, Sun F, Han SB, et al. B-RAF kinase drives developmental axon growth and promotes axon regeneration in the injured mature CNS. J Exp Med. 2014;211:801–14. 10.1084/jem.20131780 PMC4010899 24733831

[105] Mazzoni IE, Saïd FA, Aloyz R, Miller FD, Kaplan D. Ras regulates sympathetic neuron survival by suppressing the p53-mediated cell death pathway. J Neurosci. 1999;19:9716–27. 10.1523/JNEUROSCI.19-22-09716.1999 PMC6782948 10559381

[106] Zhong J. RAS and downstream RAF-MEK and PI3K-AKT signaling in neuronal development, function and dysfunction. Biol Chem. 2016;397:215–22. 10.1515/hsz-2015-0270 PMC4753800 26760308

[107] Heumann R, Goemans C, Bartsch D, Lingenhöhl K, Waldmeier PC, Hengerer B, et al. Transgenic activation of Ras in neurons promotes hypertrophy and protects from lesion-induced degeneration. J Cell Biol. 2000;151:1537–48. 10.1083/jcb.151.7.1537 PMC2150671 11134081

[108] Felderhoff-Mueser U, Bittigau P, Sifringer M, Jarosz B, Korobowicz E, Mahler L, et al. Oxygen causes cell death in the developing brain. Neurobiol Dis. 2004;17:273–82. 10.1016/j.nbd.2004.07.019 15474364

[109] Reichardt LF. Neurotrophin-regulated signalling pathways. Philos Trans R Soc B. 2006;361:1545–64. 10.1098/rstb.2006.1894 PMC1664664 16939974

[110] Paves H, Saarma M. Neurotrophins as in vitro growth cone guidance molecules for embryonic sensory neurons. Cell Tissue Res. 1997;290:285–97. 10.1007/s004410050933 9321690

[111] Fivaz M, Bandara S, Inoue T, Meyer T. Robust neuronal symmetry breaking by Ras-triggered local positive feedback. Curr Biol. 2008;18:44–50. 10.1016/j.cub.2007.11.051 18158244

[112] Raudzus F, Schöneborn H, Neumann S, Secret E, Michel A, Fresnais J, et al. Magnetic spatiotemporal control of SOS1 coupled nanoparticles for guided neurite growth in dopaminergic single cells. Sci Rep. 2020;10:22452. 10.1038/s41598-020-80253-w PMC7775457 33384447

[113] Adachi H, Morizane A, Torikoshi S, Raudzus F, Taniguchi Y, Miyamoto S, et al. Pretreatment with perlecan-conjugated laminin-E8 fragment enhances maturation of grafted dopaminergic progenitors in Parkinson’s disease model. Stem Cells Transl Med. 2022;11:767–77. 10.1093/stcltm/szac033 PMC9299512 35605097

[114] Korshunova I, Rhein S, García-González D, Stölting I, Pfisterer U, Barta A et al. Genetic modification increases the survival and the neuroregenerative properties of transplanted neural stem cells. JCI Insight. 2020;5. 10.1172/jci.insight.126268. PMC7101138 31999645

[115] Liang Y, Ågren L, Lyczek A, Walczak P, Bulte JWM. Neural progenitor cell survival in mouse brain can be improved by co-transplantation of helper cells expressing bFGF under doxycycline control. Exp Neurol. 2013;247:73–79. 10.1016/j.expneurol.2013.04.001 PMC3742733 23570903

[116] Gowing G, Shelley B, Staggenborg K, Hurley A, Avalos P, Victoroff J, et al. Glial cell line-derived neurotrophic factor-secreting human neural progenitors show long-term survival, maturation into astrocytes, and no tumor formation following transplantation into the spinal cord of immunocompromised rats. NeuroReport. 2014;25:367–72. 10.1097/WNR.0000000000000092 PMC3969154 24284956

[117] Roy NS, Cleren C, Singh SK, Yang L, Beal MF, Goldman SA. Functional engraftment of human ES cell–derived dopaminergic neurons enriched by coculture with telomerase-immortalized midbrain astrocytes. Nat Med. 2006;12:1259–68. 10.1038/nm1495 17057709

[118] Baloh RH, Johnson JP, Avalos P, Allred P, Svendsen S, Gowing G, et al. Transplantation of human neural progenitor cells secreting GDNF into the spinal cord of patients with ALS: a phase 1/2a trial. Nat Med. 2022;28:1813–22. 10.1038/s41591-022-01956-3 PMC9499868 36064599

[119] You Z, Savitz SI, Yang J, Degterev A, Yuan J, Cuny GD, et al. Necrostatin-1 reduces histopathology and improves functional outcome after controlled cortical impact in mice. J Cereb Blood Flow Metab. 2008;28:1564–73. 10.1038/jcbfm.2008.44 PMC2831087 18493258

[120] Xu X, Chua K-W, Chua CC, Liu C-F, Hamdy RC, Chua BHL. Synergistic protective effects of humanin and necrostatin-1 on hypoxia and ischemia/reperfusion injury. Brain Res. 2010;1355:189–94. 10.1016/j.brainres.2010.07.080 PMC3412340 20682300

[121] Yang S, Lee DK, Shin J, Lee S, Baek S, Kim J, et al. Nec-1 alleviates cognitive impairment with reduction of Aβ and tau abnormalities in APP/PS1 mice. EMBO Mol Med. 2017;9:61–77. 10.15252/emmm.201606566 PMC5210088 27861127

[122] Jantas D, Greda A, Golda S, Korostynski M, Grygier B, Roman A, et al. Neuroprotective effects of metabotropic glutamate receptor group II and III activators against MPP(+)-induced cell death in human neuroblastoma SH-SY5Y cells: The impact of cell differentiation state. Neuropharmacology. 2014;83:36–53. 10.1016/j.neuropharm.2014.03.019 24713472

[123] Kim D-Y, Leem Y-H, Park J-S, Park J-E, Park J-M, Kang JL, et al. RIPK1 regulates microglial activation in lipopolysaccharide-induced neuroinflammation and MPTP-induced Parkinson’s disease mouse models. Cells. 2023;12:417. 10.3390/cells12030417 PMC9913664 36766759

[124] Iannielli A, Bido S, Folladori L, Segnali A, Cancellieri C, Maresca A, et al. Pharmacological inhibition of necroptosis protects from dopaminergic neuronal cell death in Parkinson’s disease models. Cell Rep. 2018;22:2066–79. 10.1016/j.celrep.2018.01.089 PMC5842028 29466734

[125] Lei Z-N, Liu F, Zhang L-M, Huang Y-L, Sun F-Y. Bcl-2 increases stroke-induced striatal neurogenesis in adult brains by inhibiting BMP-4 function via activation of β-catenin signaling. Neurochem Int. 2012;61:34–42. 10.1016/j.neuint.2012.04.004 22521772

[126] Islam R, Ahlfors J-E, Siu R, Noman H, Akbary R, Morshead CM. Inhibition of apoptosis in a model of ischemic stroke leads to enhanced cell survival, endogenous neural precursor cell activation and improved functional outcomes. Int J Mol Sci. 2024;25:1786. 10.3390/ijms25031786 PMC10855341 38339065

[127] Shabanzadeh AP, D’Onofrio PM, Monnier PP, Koeberle PD. Targeting caspase-6 and caspase-8 to promote neuronal survival following ischemic stroke. Cell Death Dis. 2015;6:e1967. 10.1038/cddis.2015.272 PMC4670918 26539914

[128] Qiao C, Zhang L-X, Sun X-Y, Ding J-H, Lu M, Hu G. Caspase-1 deficiency alleviates dopaminergic neuronal death via inhibiting caspase-7/AIF pathway in MPTP/p mouse model of Parkinson’s disease. Mol Neurobiol. 2017;54:4292–302. 10.1007/s12035-016-9980-5 27339879

[129] Bao W, Zhou X, Zhou L, Wang F, Yin X, Lu Y, et al. Targeting miR-124/Ferroportin signaling ameliorated neuronal cell death through inhibiting apoptosis and ferroptosis in aged intracerebral hemorrhage murine model. Aging Cell. 2020;19:e13235. 10.1111/acel.13235 PMC7681046 33068460

[130] Wang Y, Niu H, Li L, Han J, Liu Z, Chu M, et al. Anti-CHAC1 exosomes for nose-to-brain delivery of miR-760-3p in cerebral ischemia/reperfusion injury mice inhibiting neuron ferroptosis. J Nanobiotechnology. 2023;21:109. 10.1186/s12951-023-01862-x PMC10041751 36967397

[131] Ma P, Li Y, Zhang W, Fang F, Sun J, Liu M, et al. Long non-coding RNA MALAT1 inhibits neuron apoptosis and neuroinflammation while stimulates neurite outgrowth and its correlation with MiR-125b mediates PTGS2, CDK5 and FOXQ1 in Alzheimer’s disease. Curr Alzheimer Res. 2019;16:596–612. 10.2174/1567205016666190725130134 31345147

[132] Nguyen LD, Wei Z, Silva MC, Barberán-Soler S, Zhang J, Rabinovsky R, et al. Small molecule regulators of microRNAs identified by high-throughput screen coupled with high-throughput sequencing. Nat Commun. 2023;14:7575. 10.1038/s41467-023-43293-0 PMC10663445 37989753

[133] Iwata R, Casimir P, Erkol E, Boubakar L, Planque M, López IMG, et al. Mitochondria metabolism sets the species-specific tempo of neuronal development. Science. 2023;379:eabn4705. 10.1126/science.abn4705 36705539

[134] Ciceri G, Baggiolini A, Cho HS, Kshirsagar M, Benito-Kwiecinski S, Walsh RM, et al. An epigenetic barrier sets the timing of human neuronal maturation. Nature. 2024;626:881–90. 10.1038/s41586-023-06984-8 PMC10881400 38297124

[135] Russo GL, Sonsalla G, Natarajan P, Breunig CT, Bulli G, Merl-Pham J, et al. CRISPR-mediated induction of neuron-enriched mitochondrial proteins boosts direct glia-to-neuron conversion. Cell Stem Cell. 2021;28:524–34. e7. 10.1016/j.stem.2020.10.015 PMC7939544 33202244

[136] Khacho M, Clark A, Svoboda DS, Azzi J, MacLaurin JG, Meghaizel C, et al. Mitochondrial dynamics impacts stem cell identity and fate decisions by regulating a nuclear transcriptional program. Cell Stem Cell. 2016;19:232–47. 10.1016/j.stem.2016.04.015 27237737

[137] Iwata R, Casimir P, Vanderhaeghen P. Mitochondrial dynamics in postmitotic cells regulate neurogenesis. Science. 2020;369:858–62. 10.1126/science.aba9760 32792401

[138] Ocampo A, Reddy P, Martinez-Redondo P, Platero-Luengo A, Hatanaka F, Hishida T, et al. In vivo amelioration of age-associated hallmarks by partial reprogramming. Cell. 2016;167:1719–33. e12. 10.1016/j.cell.2016.11.052 PMC5679279 27984723

[139] Gill D, Parry A, Santos F, Okkenhaug H, Todd CD, Hernando-Herraez I, et al. Multi-omic rejuvenation of human cells by maturation phase transient reprogramming. eLife. 2022;11:e71624. 10.7554/eLife.71624 PMC9023058 35390271

[140] Sarkar TJ, Quarta M, Mukherjee S, Colville A, Paine P, Doan L, et al. Transient non-integrative expression of nuclear reprogramming factors promotes multifaceted amelioration of aging in human cells. Nat Commun. 2020;11:1545. 10.1038/s41467-020-15174-3 PMC7093390 32210226

[141] Olova N, Simpson DJ, Marioni RE, Chandra T. Partial reprogramming induces a steady decline in epigenetic age before loss of somatic identity. Aging Cell. 2019;18:e12877. 10.1111/acel.12877 PMC6351826 30450724

[142] Mitchell W, Goeminne LJ, Tyshkovskiy A, Zhang S, Chen JY, Paulo JA, et al. Multi-omics characterization of partial chemical reprogramming reveals evidence of cell rejuvenation. eLife. 2024;12:RP90579. 10.7554/eLife.90579 PMC10959535 38517750

[143] Kim H, Cooke MJ, Shoichet MS. Creating permissive microenvironments for stem cell transplantation into the central nervous system. Trends Biotechnol. 2012;30:55–63. 10.1016/j.tibtech.2011.07.002 21831464

[144] Damle EB, Morrison VE, Cioma J, Volic M, Bix GJ. Co-administration of extracellular matrix-based biomaterials with neural stem cell transplantation for treatment of central nervous system injury. Front Neurosci. 2023;17:1177040. 10.3389/fnins.2023.1177040 PMC10225608 37255752

[145] Zvibel I, Smets F, Soriano H. Anoikis: roadblock to cell transplantation? Cell Transplant. 2002;11:621–30. 10.3727/000000002783985404 12518889

[146] Frisco-Cabanos HL, Watanabe M, Okumura N, Kusamori K, Takemoto N, Takaya J, et al. Synthetic molecules that protect cells from anoikis and their use in cell transplantation. Angew Chem Int Ed. 2014;53:11208–13. 10.1002/anie.201405829 25196666

[147] Comini G, Kelly R, Jarrin S, Patton T, Narasimhan K, Pandit A, et al. Survival and maturation of human induced pluripotent stem cell-derived dopaminergic progenitors in the parkinsonian rat brain is enhanced by transplantation in a neurotrophin-enriched hydrogel. J Neural Eng. 2024;21:024002. 10.1088/1741-2552/ad33b2 38479026

[148] Moriarty N, Cabré S, Alamilla V, Pandit A, Dowd E. Encapsulation of young donor age dopaminergic grafts in a GDNF-loaded collagen hydrogel further increases their survival, reinnervation, and functional efficacy after intrastriatal transplantation in hemi-Parkinsonian rats. Eur J Neurosci. 2019;49:487–96. 10.1111/ejn.14090 30054941

[149] Payne SL, Tuladhar A, Obermeyer JM, Varga BV, Teal CJ, Morshead CM, et al. Initial cell maturity changes following transplantation in a hyaluronan-based hydrogel and impacts therapeutic success in the stroke-injured rodent brain. Biomaterials. 2019;192:309–22. 10.1016/j.biomaterials.2018.11.020 30468998

[150] Ballios BG, Cooke MJ, Donaldson L, Coles BLK, Morshead CM, van der Kooy D, et al. A Hyaluronan-based injectable hydrogel improves the survival and integration of stem cell progeny following transplantation. Stem Cell Rep. 2015;4:1031–45. 10.1016/j.stemcr.2015.04.008 PMC4471829 25981414

[151] Wu S, Xu R, Duan B, Jiang P. Three-dimensional hyaluronic acid hydrogel-based models for in vitro human iPSC-derived NPC culture and differentiation. J Mater Chem B. 2017;5:3870–8. 10.1039/C7TB00721C PMC5536346 28775848

[152] He Z, Zang H, Zhu L, Huang K, Yi T, Zhang S, et al. An anti-inflammatory peptide and brain-derived neurotrophic factor-modified hyaluronan-methylcellulose hydrogel promotes nerve regeneration in rats with spinal cord injury. Int J Nanomed. 2019;14:721–32. 10.2147/IJN.S187854 PMC6342221 30705588

[153] Wang Y, Wei YT, Zu ZH, Ju RK, Guo MY, Wang XM, et al. Combination of hyaluronic acid hydrogel scaffold and PLGA microspheres for supporting survival of neural stem cells. Pharm Res. 2011;28:1406. 10.1007/s11095-011-0452-3 21537876

[154] Joung D, Truong V, Neitzke CC, Guo S, Walsh PJ, Monat JR et al. 3D printed stem-cell derived neural progenitors generate spinal cord scaffolds. Adv Funct Mater. 2018;28. 10.1002/adfm.201801850. PMC7319181 32595422

[155] Lilja J, Ivaska J. Integrin activity in neuronal connectivity. J Cell Sci. 2018;131:jcs212803. 10.1242/jcs.212803 29907643

[156] Park YK, Goda Y. Integrins in synapse regulation. Nat Rev Neurosci. 2016;17:745–56. 10.1038/nrn.2016.138 27811927

[157] Nikonenko I, Toni N, Moosmayer M, Shigeri Y, Muller D, Jones LS. Integrins are involved in synaptogenesis, cell spreading, and adhesion in the postnatal brain. Dev Brain Res. 2003;140:185–94. 10.1016/s0165-3806(02)00590-4 12586424

[158] Temple S. Advancing cell therapy for neurodegenerative diseases. Cell Stem Cell. 2023;30:512–29. 10.1016/j.stem.2023.03.017 PMC10201979 37084729

[159] Boato F, Guan X, Zhu Y, Ryu Y, Voutounou M, Rynne C, et al. Activation of MAP2K signaling by genetic engineering or HF-rTMS promotes corticospinal axon sprouting and functional regeneration. Sci Transl Med. 2023;15:eabq6885. 10.1126/scitranslmed.abq6885 36599003

[160] Nieuwenhuis B, Barber AC, Evans RS, Pearson CS, Fuchs J, MacQueen AR, et al. PI 3-kinase delta enhances axonal PIP3 to support axon regeneration in the adult CNS. EMBO Mol Med. 2020;12:EMMM201911674. 10.15252/emmm.201911674 PMC7411663 32558386

[161] Shabanzadeh AP, D’Onofrio PM, Magharious M, Choi KAB, Monnier PP, Koeberle PD. Modifying PTEN recruitment promotes neuron survival, regeneration, and functional recovery after CNS injury. Cell Death Dis. 2019;10:567. 10.1038/s41419-019-1802-z PMC6662832 31358730

[162] Campion TJ, Sheikh IS, Smit RD, Iffland PH, Chen J, Junker IP, et al. Viral expression of constitutively active AKT3 induces CST axonal sprouting and regeneration, but also promotes seizures. Exp Neurol. 2022;349:113961. 10.1016/j.expneurol.2021.113961 34953897

[163] Wu D, Yang P, Zhang X, Luo J, Haque ME, Yeh J, et al. Targeting a dominant negative rho kinase to neurons promotes axonal outgrowth and partial functional recovery after rat rubrospinal tract lesion. Mol Ther. 2009;17:2020–30. 10.1038/mt.2009.168 PMC2814380 19623163

[164] Patel RK, Prasad N, Kuwar R, Haldar D, Abdul-Muneer PM. Transforming growth factor-beta 1 signaling regulates neuroinflammation and apoptosis in mild traumatic brain injury. Brain Behav Immun. 2017;64:244–58. 10.1016/j.bbi.2017.04.012 28433746

[165] Wang J-J, Zhu J-D, Zhang X-H, Long T-T, Ge G, Yu Y. Neuroprotective effect of Notch pathway inhibitor DAPT against focal cerebral ischemia/reperfusion 3 h before model establishment. Neural Regen Res. 2019;14:452–61. 10.4103/1673-5374.245469 PMC6334612 30539813

[166] Dong Y, Fischer R, Naudé PJW, Maier O, Nyakas C, Duffey M, et al. Essential protective role of tumor necrosis factor receptor 2 in neurodegeneration. Proc Natl Acad Sci USA. 2016;113:12304–9. 10.1073/pnas.1605195113 PMC5087045 27791020

[167] Gao Y, Deng K, Hou J, Bryson JB, Barco A, Nikulina E, et al. Activated CREB is sufficient to overcome inhibitors in myelin and promote spinal axon regeneration in vivo. Neuron. 2004;44:609–21. 10.1016/j.neuron.2004.10.030 15541310

